# Differential Electrophysiological Responses to Odorant Isotopologues in Drosophilid Antennae^1^,^2^,^3^

**DOI:** 10.1523/ENEURO.0152-15.2016

**Published:** 2016-06-20

**Authors:** Efstathia Drimyli, Alexandros Gaitanidis, Klio Maniati, Luca Turin, Efthimios M. C. Skoulakis

**Affiliations:** 1 Division of Neuroscience, Biomedical Sciences Research Centre “Alexander Fleming,” 16672 Vari, Greece; 2 Department of Basic Sciences, School of Nursing, National and Kapodistrian University of Athens, 11527 Athens, Greece; 3 Department of Chemical Sciences, School of Chemical Engineering, National Technical University of Athens, 15780 Athens, Greece; 4 Institute of Theoretical Physics, Ulm University, 89073 Ulm, Germany

**Keywords:** antennograms, Drosophila, isotopomers, molecular vibrations, olfaction, olfactory receptors

## Abstract

Olfaction presents the ultimate challenge to molecular recognition as thousands of molecules have to be recognized by far fewer olfactory receptors. We have presented evidence that *Drosophila* readily distinguish odorants based on their molecular vibrations using a battery of behavioral assays suggesting engagement of a molecular vibration-sensing component. Here we interrogate electrophysiologically the antennae of four Drosophilids and demonstrate conserved differential response amplitudes to aldehydes, alcohols, ketones, nitriles, and their deuterated isotopologues. Certain deuterated odorants evoked larger electroantennogram (EAG) amplitudes, while the response to the normal odorant was elevated in others. Significantly, benzonitrile isotopologues were not distinguishable as predicted. This suggests that isotopologue-specific EAG amplitudes result from differential activation of specific olfactory receptors. In support of this, odorants with as few as two deuteria evoke distinct EAG amplitudes from their normal isotopologues, and this is independent of the size of the deuterated molecule. Importantly, we find no evidence that these isotopologue-specific amplitudes depend on perireceptor mechanisms or other pertinent physical property of the deuterated odorants. Rather, our results strongly suggest that Drosophilid olfactory receptors are activated by molecular vibrations differentiating similarly sized and shaped odorants *in vivo,* yielding sufficient differential information to drive behavioral choices.

## Significance Statement

Insects can behaviorally discriminate odorants from their deuterated isotopologues. It remains unclear whether discrimination occurs because olfactory receptors detect their distinct molecular vibrations or because isotopologues differ sufficiently in other properties to be perceived differentially. We report electrophysiological recordings, taken from the antennae of several Drosophilid species exposed to a range of odorant isotopologues. We find that, in almost all cases of odorants and species tested, electroantennogram amplitude, but not kinetics, differs in response to hydrogen-only and deuterated odorants. The inhibition of enzymes that prevalently catabolize odorants has no effect on the isotopologue-specific response. Hence, we conclude that, *in vivo*, isotopologue differentiation is not a perireceptor event, but likely occurs at the receptor itself.

## Introduction

Thousands of odorants are discriminated with exquisite specificity by a far smaller number of olfactory receptors (ORs). Multiple receptors are activated by particular odorants, fewer by others, and diverse odorants can activate the same OR (Buck, 2004). Therefore, ORs likely recognize multiple, potentially distinct, molecular features and functional groups of odorant molecules, including their molecular vibrations, as previously suggested (Turin, 1996; Franco et al., 2011). Odorants containing nonexchangeable deuterium in place of hydrogen ([isotopically substituted odorants (or isotopologues)] retain the shape, physical properties, and ground–state conformations of their normal counterparts (Wade, 1999). However, they are vibrationally distinct, because the C–H stretch, for example, vibrates with a frequency of 2950–3000 cm, whereas the C–D stretch at 2150 cm due to the additional neutron in deuterium (Turin, 1996; Wade, 1999; Haffenden et al., 2001). Hence, the discrimination of deuterated from deuterated (normal) isotopologues by ORs is consistent with the detection of odorant molecular vibrations. The recognition of odorant vibrational modes could provide additional discriminatory cues, which together with their size and functional groups, could contribute to the OR recognition repertoire, hence to olfactory selectivity. Behavioral experiments strongly suggest that the insect olfactory system detects molecular vibrations and uses them to drive behavioral choices (Franco et al., 2011; Bittner et al., 2012; Gronenberg et al., 2014). Importantly, *Drosophila* trained to avoid a deuterated odorant exhibit learned aversion for the chemically unrelated nitrile functional group, which, however, shares vibrational frequency with the C–D stretch at 2150 cm (Franco et al., 2011).

Alternative explanations to vibration-based isotopologue discrimination have been suggested because deuterated compounds are slightly heavier due to the extra neutrons. This could result in differential isotopologue diffusion through the sensillar lymph to the ORs, through differential isotopologue affinity for ORs, or through odorant binding proteins (OBPs). OBPs are small proteins in the lymph surrounding the receptor thought to be involved in odorant transport (Heydel et al., 2013; Leal, 2013). In addition, enzymatic processing in the OR-surrounding lymph has been suggested as a contributor to isotopologue differentiation (Block et al., 2015a, b) because of potential differences in their chemistry. Such activities are largely mediated by P450 cytochromes (CYPs) in *Drosophila* (Wang et al., 1999; Younus et al., 2014) and vertebrate olfactory organs (Schilling et al., 2010). These considerations prompted us to address the potential contribution of the enzymatic and kinetic effects of odorant size, functional groups, and other parameters to differential isotopologue detection. Furthermore, because of these considerations we addressed these questions *in situ*, rather than in heterologous expression systems, where lack of perireceptor enzymes and the OBP milieu (Heydel et al., 2013; Leal, 2013) could compromise isotopologue discrimination.

Given the mammalian complexity, with large numbers of ORs and millions of olfactory sensory neurons (OSNs; Godfrey et al., 2004; Malnic et al., 2004; Patel and Pinto, 2014), we used *Drosophila* because it is well suited for an *in vivo* approach, can differentiate isotopologues (Franco et al., 2011; Bittner et al., 2012), and follows the same organizational principles as vertebrate olfactory systems (Leal, 2013; Martin et al., 2013). *Drosophila melanogaster* expresses 62 heterodimeric ORs residing within broadly stereotypically distributed sensilla in the fly antenna (Hallem et al., 2004, 2006; Tunstall and Warr, 2012). *Drosophila* ORs contain an odorant engaging variable subunit and a common coreceptor encoded by the *Orco* gene (Larsson et al., 2004; Sato et al., 2008; Wicher et al., 2008), and this is a major difference with the monomeric mammalian receptors. In addition, although fly ORs also contain seven transmembrane domains, they are distinct from the mammalian receptors, which belong to the typical G-protein-coupled receptor (GPCR) family (Buck, 2004). At least 14 additional receptors related to glutamate ionotropic receptors (IRs) are also present in the antenna and often coreside with particular ORs within specific OSNs (Abuin et al., 2011; Rytz et al., 2013).

Although *Drosophila* ORs differ structurally from their mammalian counterparts, they offer the major advantage of being readily amenable to direct activity measurements of single OSNs or populations of OSNs *in vivo*. Electroantennograms (EAGs) probe the sum of receptor activities in the antenna, or at least in the broader vicinity of the electrode probe (Ayer and Carlson, 1991, 1992). We assessed the physiological response of *Drosophila* antennae to multiple odorant isotopologues in live animals and generalized our findings to other species within the genus *Drosophila* covering the 40 million years that separate *D. melanogaster* from *Drosophila virilis* (Ashburner et al., 1981; Markow and O’Grady, 2006) using both electrophysiological (i.e., EAGs) and behavioral approaches.

## Materials and Methods

### EAG measurements

Recordings were obtained from immobilized 3- to 10-d-old females that were maintained at 20°C and 40–60% relative humidity. Each fly was immobilized by insertion into the narrow end of a truncated plastic yellow pipette tip, with the head facing upward protruding from the open end and was then placed under a dissecting microscope (model SZX16, Olympus). The antenna was lifted and fixed on a coverslip with the aid of a glass micropipette tip. Recording and reference glass microelectrodes filled with 0.17 m NaCl (Venard and Pichon, 1984) were placed in the third antennal segment and the eye (ground), respectively. The recording electrode was placed in the middle of the dorsoventral axis of the inner-facing (away from the eyes) lateral side of the 3rd antennal segment near the area that the antennal nerve exits. The signal was amplified through a patch-clamp amplifier (MultiClamp 700B, Molecular Devices), fed into a computer via a 16 bit analog-to-digital converter (Digidata 1440A, Molecular Devices) with a sampling rate of 4 kHz and analyzed with pCLAMP version 10.3 software (Molecular Devices).

The stimulus was applied using a controller (Stimulus Controller CS-55, Syntech) generating a continuous, humidified, charcoal-filtered air flow of 1.0 L/min to which the odorant stimulus was added at a flow rate of 0.5 L/min. The stimulus was carried in a continuous airstream and was interchanged with plain air automatically to avoid mechanical stimulation of the antennae. The airstream was directed at the fly through a plastic tube (1 cm diameter), which was fixed in position by a manipulator such that its output was ∼1 cm away from the head of the fly.

All odorants were tested for purity by gas chromatography (see [Table T2][Table T3][Table T5]) after the completion of each experimental set using them to ascertain a lack of possible degradation during the experimental protocol. Each odorant was diluted in isopropyl myristate (IPM; Sigma-Aldrich), and 20 μl of the stimulus solution were applied on a strip of filter paper (0.3 × 5 cm; Whatman, GE Healthcare), which was then inserted into a Pasteur pipette. This was then attached to the stimulus carrying tubing of the CS-55. Each fly was stimulated twice with a single isotopologue pair (normal and deuterated). The duration of the stimulus was 0.5 s, and the order in which the isotopologues were presented was random.

To eliminate variation in the amount of odorant present in the first stimulus, the EAG amplitude was measured from the prestimulation baseline to the maximal odorant-induced polarization only for the second stimulus with each isotopologue. The peak amplitude difference was calculated for each pair of odorants given to a fly and was reported as the means of absolute differences with values above zero representing cases where the response of the h-odorant was higher than that of its isotopologue, and less than zero if vice versa. Differences in response to the two isotopologues for each fly were evaluated by paired sample *t* tests (Excel).

Rise-time values were calculated as the time required to achieve two-thirds of the maximal amplitude, whereas fall time values were measured as the time necessary to recover to one-third of the maximal amplitude after stimulation, as described previously (Alcorta, 1991). Differences were evaluated for each fly by paired-sample *t* tests (Excel).

The following odorants were used: benzaldehyde (BNZ; Sigma-Aldrich and Fluka Analytical), 2-hexanone (HEN; Sigma-Aldrich), 1-hexanol (HEL; Fluka Analytical and Lluch Essence), benzonitrile (BNL; Sigma-Aldrich), ethanol (Fisher), 1-pentanol (Ernesto Ventos), 1-octanol (Sigma-Aldrich), acetophenone (ACP; Puriss grade; Fluka Analytical), and benzoic acid (Sigma-Aldrich). All deuterated odorants (benzaldehyde-d6, 2-hexanone-1,1,1,3,3-d5, *n*-hexyl-1,1-d2 alcohol, *n*-hexyl-5,5,6,6,6-d5 alcohol, *n*-hexyl-d13 alcohol, benzonitrile-d5, ethyl alcohol-d6, 1-pentyl-d11 alcohol, 1-octyl-d17 alcohol, aceto-d3-phenone, acetophenone-2',3',4',5',6'-d5, acetophenone-d8, and d5-benzoic acid) were from CDN Isotopes.

### Enzyme inhibition assays

*Drosophila* P450 CYPs (Wang et al., 1999; Younus et al., 2014) were inhibited with piperonyl butoxide (PBO) based on the protocol of Wang et al. (2013) and the involvement of these enzymes in methanol detoxification.

#### Lethality assays

Ten 3- to 5-d-old adult flies were placed in glass vials (75 × 26 mm) containing 3 ml of minimal food (1% agar and 2% sucrose), the indicated amount of pure methanol (percentage, v/v), and 30 μl of a 1:3 PBO-acetone solution or acetone vehicle alone, as indicated, applied on a 0.5 × 5 mm piece of Whatman paper. The number of dead flies was scored after 18 h at 25°C, and the experiment was repeated eight independent times.

#### Physiology assays

After 18 h of exposure to PBO, flies were taken from glass vials and were mounted for EAGs as described above. For recovery experiments, flies exposed to PBO for 18 h were transferred without anesthesia to vials containing minimal food without drug and were assayed for EAG responses 18 h later. Control flies were treated with vehicle alone (acetone) on minimal media for the same time as experimental animals.

### Behavioral assays

All *Drosophila* species were reared and maintained on standard fly food (Acevedo et al., 2007) at 25°C, except for *Drosophila pseudoobcura*, which were reared and maintained at 22°C to account for their Alpine habitat (Kuntz and Eisen, 2014). Handling before and during the behavioral experiments was as described previously (Franco et al., 2011). Conditioning for *D. pseudoobscura* was performed at 24°C and 70% humidity. The glass vials containing the odorants were sealed with a rubber plug that allowed an air stream of 500 ml/min to pass through. In order for a constant surface area to be maintained, all odorants were diluted to a total of 1 ml of IPM. The amounts of odorants used were determined empirically so as to evoke a similar naive responses (Franco et al., 2011) and are shown in Table 1. The amounts below refer to those used for training and testing with each isotopologue such that a concentration of 5 μl/ml was used for training *D. melanogaster* with h-HEL, and a concentration of 5 μl/ml for each was used for testing. Conversely, a concentration of 1 μl/ml was used for training *D. melanogaster* with d13-HEL, and a concentration of 1 μl/ml was used for each isotopologue for testing.

**Table 1:** Concentrations of hexanol isotopologues used for conditioning experiments

	h-hexanol+IPM	d13-hexanol+IPM
*D. melanogaster* (*w^1118^*)	5 μl/ml	1 μl/ml
*D. simulans*	2 μl/ml	5 μl/ml
*D. pseudoobscura*	2 μl/ml	2 μl/ml
*D. virilis*	20 μl/ml	20 μl/ml
*D. melanogaster* (Canton-S)	2 μl/ml	2 μl/ml

Groups of 40–60 flies were placed into the training arm of a standard olfactory conditioning maze (Franco et al., 2011) and were presented for 1 min with the odorant while receiving 12 electric footshocks of 90 V DC each lasting 1.2 s. The training odor was then cleared from the training tube with 15 s of room air. Subsequently, the flies were transferred to the choice point of the maze where an air stream carrying an odorant met with another that passed over IPM only. The flies then had 90 s to move away or toward the test odorant, which was either the same as the training odorant or its isotopologue. The assay was executed in groups of three trials starting with naive avoidance of an odorant versus the solvent (IPM)-scented air. Another group of flies was tested with a test isotopologue identical to the one used for training (odor plus shock). Finally, for the third group of flies, the training odorant was the other isotopologue of the test odorant. After every trial, the flies were collected and counted, and a distribution index was calculated by subtracting the number of flies that did not avoid the test odorant from those that did, divided by the total counted from each trial. Results were analyzed parametrically using planned comparisons (least-squares method) and the statistical program JMP (SAS Institute).

## Results

### Differential EAG responses to isotopologues

Receptors may be activated or inhibited by odorants (Hallem and Carlson, 2006; Carey and Carlson, 2011), but EAGs do not differentiate between such events (Alcorta, 1991). Rather, the EAG amplitude reflects the sum of receptor activities as voltage changes upon odorant exposure. If the engagement of odorant isotopologues results in differential patterns of OR activation and inhibition, it will yield differential voltage changes reflected by distinct EAG properties (Alcorta, 1991; Ayer and Carlson, 1992).

We used this approach to interrogate the overall electrophysiological response of *Drosophila* antennae to previously described (Franco et al., 2011) and new odorant isotopologue pairs in three concentrations (0.01%, 0.1%, and 1% v/v). Initially, we used isotopologue pairs of common functional groups representing simple alcohols (HEL), aromatics and aldehydes (BNZ), simple ketones (HEN), and nitriles (BNL). The purity of all odorants was ascertained by gas chromatography (Tables 2, 3; also see Table 5). The recording electrode was placed in approximately the same location of the midpoint along the dorsoventral axis of the inner lateral side of the antenna in all animals tested. Individual flies were challenged with isotopologue pairs delivered in random order (see Materials and Methods). We avoided using acids (i.e., acetic acid) because these activate IRs preferentially (Benton et al., 2009; Rytz et al., 2013). Therefore, with the odorants used we are assaying primarily the response of the OR class of receptors.

**Table 2: T2:** Purity of normal and deuterated isotopologues of the standard odorant set

h-1-Hexanol (Fluka Analytical)							d2-1-Hexanol						
Peak #	Ret time (min)	Type	Width (min)	Area (pA*s)	Height (pA)	Area %	Peak #	Ret time (min)	Type	Width (min)	Area (pA*s)	Height (pA)	Area %
1	4.314	BB	0.0273	2.69500	1.63299	0.04475	1	4.622	BB	0.0301	4.95090e-1	2.61661e-1	0.00676
2	5.653	BB	0.0369	7.43677e-1	3.22039e-1	0.01235	2	4.847	BB	0.0326	2.15994	1.02467	0.02950
3	**6.214**	**BB**	**0.0578**	**6012.02588**	**1353.56238**	**99.82841**	3	5.638	BV	0.0310	1.22029	6.18148e-1	0.01667
4	14.426	BB	0.1929	6.89491	4.49524e-1	0.11449	4	**6.277**	**BB**	**0.0710**	**7312.0483**	**1446.4130**	**99.88262**
							5	6.953	BB	0.0404	1.71584	5.82760e-1	0.02344
							6	9.090	BB	0.0251	8.65162e-1	5.27801e-1	0.01182
							7	11.268	BB	0.0280	1.23281	7.18195e-1	0.01684
							8	11.420	BB	0.0258	3.41531e-1	2.24189e-1	0.00467
							9	13.871	BB	0.0324	5.62412e-1	2.92345e-1	0.00768
d5-1-Hexanol							d13-1-Hexanol (lot X241P13)						
Peak #	Ret time (min)	Type	Width (min)	Area (pA*s)	Height (pA)	Area %	Peak #	Ret time (min)	Type	Width (min)	Area (pA*s)	Height (pA)	Area %
1	2.904	BB	0.0257	1.00243	6.49066e-1	0.01407	1	3.645	BB	0.0328	4.77799e-1	2.67615e-1	0.00709
2	4.796	BB	0.0339	6.67262	3.27978	0.09365	2	4.734	VB	0.0309	12.45923	6.33606	0.18490
3	5.215	BB	0.0326	2.84580	1.35165	0.03994	3	5.429	BB	0.0496	3.09593	8.68688e-1	0.04594
4	**6.232**	**BV**	**0.0739**	**7097.00391**	**1337.32166**	**99.60153**	4	5.695	BB	0.0364	8.49879e-1	3.48745e-1	0.01261
5	6.688	VB	0.0422	5.86693	2.26736	0.08234	5	**6.127**	**BB**	**0.0810**	**6720.69971**	**1285.26160**	**99.73692**
6	7.574	BB	0.0327	8.28919	3.91570	0.11633	6	7.432	BB	0.0368	8.44513e-1	3.97840e-1	0.01253
7	8.113	BB	0.0335	9.32212e-1	5.06874e-1	0.01308							
8	10.646	BB	0.0317	2.78344	1.65248	0.03906							
h-Benzaldehyde (Sigma)							d6-Benzaldehyde (lot H468P23)						
Peak #	Ret time (min)	Type	Width (min)	Area (pA*s)	Height (pA)	Area %	Peak #	Ret time (min)	Type	Width (min)	Area (pA*s)	Height (pA)	Area %
1	2.083	BB	0.0214	2.73390e-1	2.07438e-1	0.00248	1	2.702	BB	0.0403	4.31283	1.78300	0.05008
2	2.445	BB	0.0212	8.67375e-1	6.65289e-1	0.00788	2	**8.869**	**BB**	**0.0718**	**8558.87891**	**1929.26196**	**99.39384**
3	5.213	BB	0.0273	3.35924e-1	2.02958e-1	0.00305	3	10.612	BB	0.0438	1.06592	4.46972e-1	0.01238
4	**8.686**	**BB**	**0.0668**	**1.08576e4**	**2400.08643**	**98.68858**	4	12.554	BB	0.0410	9.16474	5.10110	0.10643
5	9.365	BB	0.0290	3.97409	2.20585	0.3612	5	13.386	BB	0.0584	5.53546	1.52484	0.06428
6	10.762	BB	0.0309	2.21929	1.13301	0.02017	6	15.041	BB	0.0461	12.58218	4.87559	0.14612
7	19.732	BBA	0.0395	136.61024	50.65681	1.24170	7	19.918	BB	0.0484	19.53590	7.04030	0.22687
h-Benzoic acid							d5-Benzoic acid						
Peak #	Ret time (min)	Type	Width (min)	Area (pA*s)	Height (pA)	Area %	Peak #	Ret time (min)	Type	Width (min)	Area (pA*s)	Height (pA)	Area %
1	**19.730**	**BBA**	**0.0439**	**85.00277**	**29.25834**	**1.00e2**	**1**	**19.658**	**BBA**	**0.0395**	**284.06458**	**105.29233**	**1.00e2**
h-2-hexanone							d5-2-hexanone						
Peak #	Ret time (min)	Type	Width (min)	Area (pA*s)	Height (pA)	Area %	Peak #	Ret time (min)	Type	Width (min)	Area (pA*s)	Height (pA)	Area %
1	2.122	VV	0.0172	1.22825	1.17401	0.01777	1	2.567	BB	0.0252	3.98876	3.51804	0.05061
2	**3.393**	**BV**	**0.0410**	**6898.30908**	**2597.11719**	**99.81266**	2	2.792	BV	0.0310	4.36951e-1	2.68910e-1	0.00554
3	7.690	BB	0.0329	3.76977	1.76718	0.05455	3	**3.342**	**VB**	**0.0505**	**7864.65332**	**2381.03467**	**99.79115**
4	10.191	BB	0.0277	7.94950	4.47342	0.11502	4	3.766	BB	0.0427	11.39421	4.32301	0.14458
							5	4.857	BB	0.0323	6.40037e-1	3.06994e-1	0.00812
h-Benzonitrile							d5-Benzonitrile						
Peak #	Ret time (min)	Type	Width (min)	Area (pA*s)	Height (pA)	Area %	Peak #	Ret time (min)	Type	Width (min)	Area (pA*s)	Height (pA)	Area %
1	**10.234**	**BB**	**0.0557**	**9508.75098**	**2276.43311**	**1.000e2**	1	4.644	BB	0.0344	2.18002	1.13441	0.01571
							2	5.058	BB	0.0351	4.69176e-1	2.36558e-1	0.00338
							3	6.316	BB	0.0311	18.41002	9.86647	0.13265
							4	7.883	BB	0.0400	2.80853	1.02508	0.02024
							5	**10.188**	**BB**	**0.0808**	**1.38512e4**	**2657.15698**	**99.80494**
							6	15.243	BB	0.0389	6.83049e-1	2.95831e-1	0.00492
							7	19.561	BB	0.0578	2.51985	7.02758e-1	0.01816

Results from gas chromatograms of normal, partially deuterated, and perdeuterated odorants used in the standard odorant set and relevant related odorants. The percentage (Area %) of the total that constitutes the main species within each preparation is shown in bold, as are those of all other species. For the hexanol isotopologues, the purity of all preparations was >99.6%. Contaminants varied with minimal overlap among the isotopologues and with the most abundant contaminant at 0.1–0.2%, but most others are at least 10-fold lower. Normal benzaldehyde purity was 98.7%, but the most abundant contaminant was benzoic acid (peak 7), while the contribution of others was negligible. Similarly, for d6-BNZ with 99.4% purity the most abundant contaminant was d5-benzoic acid (peak 7), as indicated by mass spectroscopy (data not shown). Solid normal and deuterated benzoic acid were dissolved in ethanol to generate a 1% solution, which did not show additional contaminants (benzoic acid isotopologues at 19.730 and 19.658 Ret times). Both hexanone isotopologues exhibited over 99.8% purity, while the contribution of contaminants was negligible. Normal benzonitrile was totally pure, while the deuterated isotopologue was 99.8% pure with negligible contribution from contaminants. Ret time, retention time; BB, Baseline to baseline; BV, Baseline to valley; VB, Valley to baseline; VV, Valley to valley.

**Figure 1.**
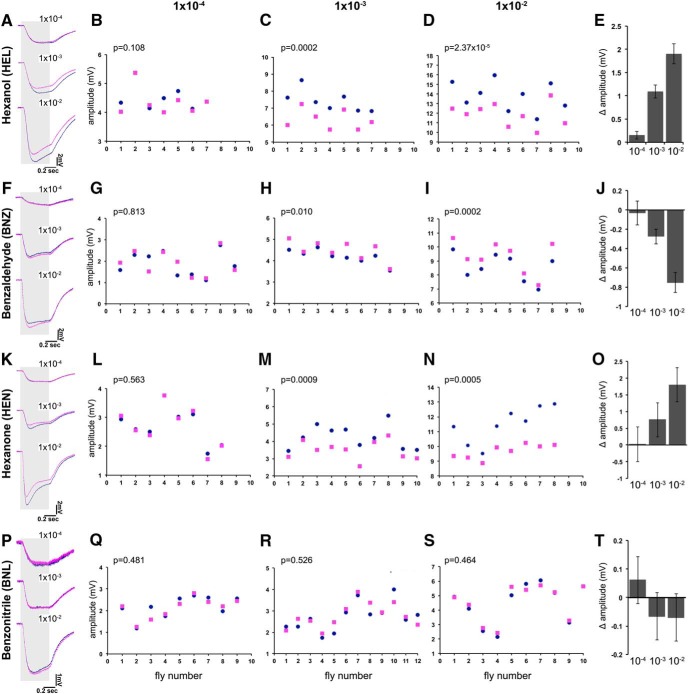
Isotopologue-specific EAG responses. Average EAG traces elicited by three concentrations of the normal (dark blue) or deuterated (magenta) isotopologues of four representative odorants are shown in ***A***, ***F***, ***K***, and ***P***. The shaded area indicates the timing and duration of odorant stimulation, while the scale is shown in the bottom right of each trace. The actual amplitude values elicited by the normal or deuterated isotopologues of HEL (***B***, ***C***, ***D***), BNZ (***G***, ***H***, ***I***), HEN (***L***, ***M***, ***N***), and BNL (***Q***, ***R***, ***S***) are represented by pairs of blue dots and magenta squares, respectively, shown for each individual, with the number of animals tested indicated in the abscissa as “fly number.” The ordinate scales have been adjusted to allow maximal resolution. The significance of isotopologue-specific amplitude differences were evaluated per fly using paired sample *t* tests and is indicated on the top left of each panel. Quantification of the collective differential response per odorant dilution was achieved by subtracting the absolute values of the h-odorant amplitude from that of the d-odorant for each fly tested, and the mean (Δ amplitude) ±SEM are shown in (***E***, ***J***, ***O***, and ***T***). Therefore, the probabilities that Δ amplitude per odorant dilution is different than 0 are the same as shown on the respective panels. The actual Δ amplitude values and their SEMs are summarized as follows:

Average EAG traces from four isotopologue pairs at three different concentrations are shown in Figure 1, A, F, K, and P. It is evident that the overall structure and details of the traces are distinct for each odorant, as expected; but, except for the EAG amplitude, they were similar for all odorant isotopologue pairs. Maximal amplitudes were consistently different between isotopologues at the lower dilutions, and we concentrated on that aspect for this study. Interestingly, h-HEL always evoked larger EAG amplitudes than d-HEL (Fig. 1C,D), and the same for HEN isotopologues (Fig. 1 M,N) at the 10^−2^ and 10^−3^ dilutions. In contrast, BNZ isotopologues yielded the opposite result with d6-BNZ, evoking a much larger response than the normal odorant in the same dilutions (Fig. 1H,I). Differences in response amplitude were not apparent at the lowest odorant concentration (10^−4^), for all odorants tested, potentially reflecting a loss of isotopologue selectivity at the higher dilutions.

**Table:** 

Dilution	Mean h-	SEM	Mean d-	SEM
HEL 10^−2^	13.7872	0.5066	11.8815	0.4079
HEL 10^−3^	7.4245	0.2400	6.3346	0.2194
HEL 10^−4^	4.5079	0.1636	4.3553	0.1808
BNZ 10^−2^	8.5526	0.3489	9.3040	0.4044
BNZ 10^−3^	4.2038	0.1121	4.4785	0.1531
BNZ 10^−4^	1.8785	0.1916	1.9165	0.1940
HEN 10^−2^	11.4822	0.4242	9.6779	0.1681
HEN 10^−3^	4.2479	0.2185	3.4943	0.1723
HEN 10^−4^	2.7123	0.2251	2.6894	0.2495
BNL 10^−2^	4.4556	0.4427	4.5258	0.4009
BNL 10^−3^	2.7186	0.1895	2.7848	0.1660
BNL 10^−4^	2.1769	0.1641	2.1159	0.1596

To quantify these isotopologue-specific EAG differences and to normalize the obvious interanimal variability, we subtracted the absolute maximal amplitude elicited by the deuterated odorant from that of its normal counterpart per animal. We then estimated the means of those differences per isotopologue pair (Δ amplitude), which, along with their SEMs, are shown in Figure 1, E, J, O, and T. Positive Δ amplitude values arise when the EAG for the normal odorant is larger than that of its deuterated isotopologue (Fig. 1E,O), whereas the converse yields negative values (Fig. 1J). If the response to the two isotopologues is similar or identical, their Δ amplitude would be zero. Because isotopologues are not expected to yield differences (Keller and Vosshall, 2004; Block et al., 2015a, b), we used this as a null hypothesis in testing for significance. Similar results for hexanol and benzaldehyde isotopologues were obtained with the Canton-S strain of *D. melanogaster* (data not shown), indicating that these responses are typical of the species and are not strain specific.

The C≡N and C–D bonds shares the ∼2150 cm vibrational frequency (Franco et al., 2011). Consequently, deuterated BNL contains both components that share the 2150 cm vibration, while the normal odorant contains only that of the C≡N bond. If the differential amplitudes depended on molecular vibrations and not the mere presence of deuterium, then the BNL isotopologues would be expected to yield similar EAGs. In fact, average traces were nearly indistinguishable at all dilutions tested (Fig. 1P), and the peak amplitudes were not significantly different at all dilutions (Fig. 1Q–S). This is further illustrated by all of the Δ amplitudes (Fig. 1T), which were not different from zero. Collectively then and in accord with predictions based on calculations, three of the four isotopologue pairs yielded differences in EAG responses, while the fourth did not, as expected. This is not consistent with the null hypothesis expecting identical responses from structurally identical molecules and agrees with prior reports (Franco et al., 2011; Bittner et al., 2012; Gane et al., 2013; Gronenberg et al., 2014) and with the hypothesis that molecular vibrations play a role in differentiating isotopologues.

Impurities are unlikely to account for these differences because hexanol and hexanone isotopologues were of very high purity (Table 2). Furthermore, deuterated benzaldehyde, which elicits higher amplitudes, is purer than its normal isotopologue (Table 2). This is not consistent with the notion that higher EAG amplitudes may result from contaminants activating additional ORs. In fact, the main benzaldehyde contaminant was benzoic acid, as mass spectroscopy identified (data not shown). Although counterintuitive given the higher EAG amplitudes from d6-BNZ, we investigated whether the presence of benzoic acid could account for the larger amplitude. We obtained pure benzoic acid isotopologues (Table 2), which yielded characteristically low signals (mean EAG amplitude for normal benzoic acid, 0.122 ± 0.221 mV), even at a much higher concentration (10^−3^) than present in BNZ samples (estimated from the gas chromatographs at <10^−4^). EAGs evoked by d5-benzoic acid were slightly higher than those from the normal compound (mean EAG amplitude, 0.096 ± 0.174 mV), with Δ amplitude not significantly different from 0 (Δ amplitude, 0.026 ± 0.273 mV; *p* = 0.927). Therefore, the EAG amplitude differences of BNZ isotopologues are not attributable to the main contaminants in the preparations. In support of this, normal 2-hexanone yielded higher EAG amplitudes (Fig. 1M,N), although the deuterated isotopologues had an additional impurity expected to yield larger EAG amplitudes if contaminants were determinants of the differential isotopologue response. Importantly, the fact that responses are higher at least for d6-benzaldehyde (Fig. 1H–J), indicates that evaporation rates, potentially reduced for the slightly heavier deuterated odorants, are unlikely to be important for the observed differential effects.

**Figure 2.**
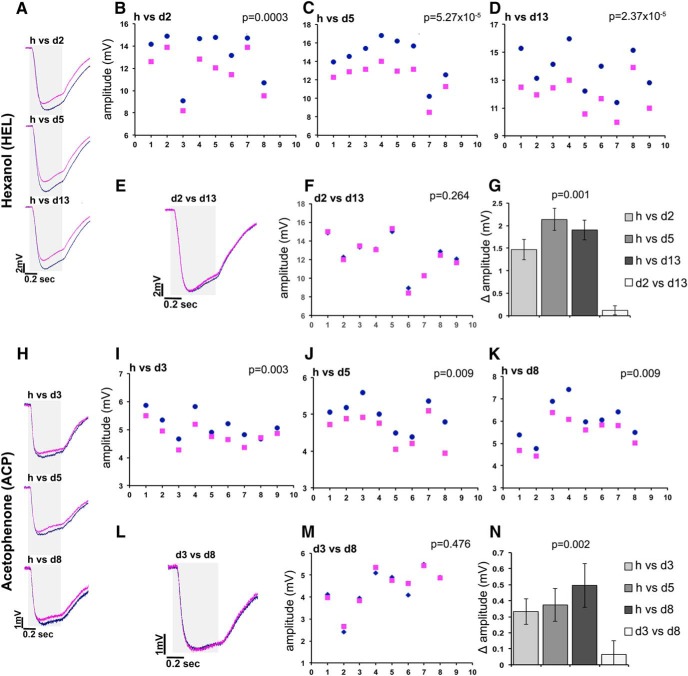
The degree of deuteration does not affect the isotopologue-specific differential response. Average EAG traces of normal (dark blue) vs deuterated (magenta) odorants are shown in ***A*** and ***H***. When two deuterated isotopologues are compared (***E***, ***L***), the blue trace corresponds to the least deuterated species. The gray area on the left side of the traces indicates the timing and duration of odorant stimulation, while the scale is shown in the bottom right of each trace. ***A–C***, Raw amplitudes elicited in response to normal and d2-hexanol (***B***), d5-hexanol (***C***), and d13-hexanol (***D***). The ordinate scales have been adjusted to allow maximal resolution. The number of flies tested with each isotopologue pair is shown in the abscissas, with each pair of blue dots and magenta squares representing the responses from single flies. Similarly, raw amplitudes in response to the di-deuterated hexanol (dark blue diamonds) vs the perdeuterated odorant (magenta squares) are shown in ***F***. The significance of isotopologue-specific amplitude differences was evaluated per fly using paired sample *t* tests and is indicated on the top right of each panel. These differential responses are quantitatively represented in ***G***, and ANOVA indicated significant differences (*F*_(3,32)_ = 17.739, *p* < 0.0001), which were revealed by least square means (LSM) contrast analysis to be due to the difference of the d2 vs d13 Δ amplitude (open bar) compared with the other three (*p* = 0.0006, *p* < 0.0001, and *p* < 0.0001, respectively, in order of increasing deuteration). In contrast, comparing the Δ amplitudes of each partially deuterated odorant over the normal isotopologue with each other did not reveal significant differences (h/d2 vs h/d13, *p* = 0.087; h/d2 vs h/d5, *p* = 0.110; h/d5 vs h/d13, *p* = 0.940). ***I–K***, Similarly, raw amplitudes elicited in response to normal and d3-acetophenone (***I***), d5-acetophenone (***J***), and d8-acetophenone (***K***). The ordinate scales have been adjusted to allow maximal resolution, while the number of flies tested with each isotopologue pair is shown in the abscissas. Each pair of blue dots and magenta squares represents the responses from single flies. Raw amplitudes in response to the d3-acetophenone (dark blue diamonds) vs the perdeuterated odorant (magenta squares) are shown in ***M***. The significance of isotopologue-specific amplitude differences were evaluated per fly using paired sample *t* tests and is indicated on each panel. These differential responses are quantitatively represented in ***N***, and ANOVA indicated significant differences (*F*_(3,32)_ = 6.331, *p* = 0.002), which were revealed by LSM contrast analysis to be due to the difference of the d3 vs d8 Δ amplitude (open bar) from the other three (*p* = 0.0075, *p* = 0.0017, and *p* = 0.0005, respectively, in order of increasing deuteration). In contrast, comparing the Δ amplitudes of each partially deuterated odorant over the normal isotopologue with each other did not reveal significant differences (h/d3 vs h/d5, *p* = 0.438; h/d3 vs h/d8, *p* = 0.245; and h/d5 vs h/d8, *p* = 0.7287).

To further ascertain that contaminants were not the source of the amplitude differences, we obtained normal odorants from different sources and different lots of their deuterated isotopologues. For emphasis, the purity profiles of the new odorants are presented separately on Table 3 and were in fact different than the original set (Table 2). For example, h-1-HEL in Table 3 contains a lot more trace impurities than that from Fluka (Table 2). In contrast, lot X421P8 of d13-1-HEL (Table 3) contains fewer impurities than the original (lot X241P13; Table 2), although from the same source (CDN Isotopes). Similar purity profile differences characterized the different lots and sources of benzaldehyde (compare Tables 2, 3) and acetophenone isotopologues (Table 3; see also Table 5). Results from the new independent odorant set are summarized in Table 4. Despite the differences in trace impurities from the previously used odorants, similar isotopologue-specific differences in amplitude, albeit somewhat different in absolute value, were observed with the different lots of HEL and BNZ (Table 4). Normal HEL contains fewer impurities than d13-HEL (Table 2) in the lots surveyed in Fig. 1. However, an independent lot of h-HEL containing more impurities (Table 3) still yielded amplitudes larger than its purer deuterated isotopologue (Table 4). A similar effect was uncovered for ACP isotopologues (Table 4, Fig. 2N). For BNZ, where the deuterated isotopologue elicits larger EAG amplitude, increased impurities in the d6-BNZ isotopologue (Table 3) still resulted in significantly different negative Δ amplitude (Table 4, −0.628; Fig. 1, −0.752) for the 10^−2^ dilution. Therefore, the amplitude differences between isotopologues remain significant irrespective of isotopologue source and lot. These results are discordant with the notion that impurities underlie the isotopologue-specific differential amplitudes. Hence, contaminating trace impurities cannot account for the differential response of the *D. melanogaster* antenna to odorant isotopologues.

**Table 3: T3:** Purity of independent lots and sources of normal and deuterated isotopologues

h-1-Hexanol (Lluch Essence)								d13-1-Hexanol (X421P8)						
Peak #	Ret time (min)	Type	Width (min)	Area (pA*s)	Height (pA)	Area %		Peak #	Ret time (min)	Type	Width (min)	Area (pA*s)	Height (pA)	Area %
1	2.196	BB	0.0369	5.72620	2.58805	0.10832		1	3.327	BB	0.0345	9.75216	6.15056	0.15369
2	2.569	BV	0.0214	5.22487	4.06881	0.09884		2	4.297	BB	0.0433	15.99059	8.01434	0.25201
3	2.726	VV	0.0331	4.29227	2.37241	0.08120		3	**5.572**	**BB**	**0.0964**	**6313.49365**	**1076.56616**	**99.49862**
4	2.856	VV	0.0308	2.58679	1.32312	0.04893		4	6.808	BB	0.0544	3.50448	1.18921	0.05523
5	3.148	VV	0.0475	3.80892	1.12580	0.07205		5	12.926	BB	0.0764	2.56706	5.30815e-1	0.04046
6	3.408	VB	0.0350	3.19802	1.62208	0.06050								
7	4.004	BB	0.0818	2.04721	3.23609e-1	0.03873								
8	5.228	BB	0.0435	3.19114	1.18053	0.06037								
9	**5.738**	**BB**	**0.0815**	**5256.20166**	**1146.01575**	**99.43107**								
h-Benzaldehyde (Fluka Analytical)								d6-Benzaldehyde (X261P20)						
Peak #	Ret time (min)	Type	Width (min)	Area (pA*s)	Height (pA)	Area %		Peak #	Ret time (min)	Type	Width (min)	Area (pA*s)	Height (pA)	Area %
1	2.375	BB	0.0224	6.39770e-1	4.53907e-1	0.00680		1	2.377	BB	0.0267	5.28992e-1	3.69073e-1	0.00567
2	**8.249**	**BB**	**0.0506**	**8977.63086**	**2349.54297**	**95.44216**		2	8.105	BV	0.0544	1.22637	3.22554e-1	0.01314
3	8.921	BB	0.0426	9.60250	3.23823	0.10209		3	**8.307**	**VB**	**0.0731**	**8838.80566**	**2099.49609**	**94.72651**
4	10.373	BB	0.0419	8.93681e-1	3.07697e-1	0.00950		4	8.937	BB	0.0342	14.52237	7.00919	0.15564
5	12.829	BB	0.0340	6.93815e-1	3.11654e-1	0.00738		5	10.126	BB	0.0512	1.07901	3.21227e-1	0.01156
6	19.153	BB	0.0427	416.89743	139.98267	4.43208		6	11.935	BB	0.0376	1.19985	5.46938e-1	0.01286
								7	12.754	BB	0.0581	2.19288	6.07426e-1	0.02350
								8	14.382	BB	0.0350	1.63216	7.62619e-1	0.01749
								9	19.128	BB	0.0530	469.68039	133.57043	5.03362
h-Acetophenone (Puriss grade, Fluka Analytical)								d8-Acetophenone (G466P32)						
Peak #	Ret time (min)	Type	Width (min)	Area (pA*s)	Height (pA)	Area %		Peak #	Ret time (min)	Type	Width (min)	Area (pA*s)	Height (pA)	Area %
1	9.530	BB	0.0452	1.14808	3.80043e-1	0.01397		1	7.637	BB	0.0381	1.46471	6.08103e-1	0.01922
2	9.819	BB	0.0802	1.13668	2.06503e-1	0.01383		2	8.188	BB	0.0365	1.16085	4.74491e-1	0.01523
3	**10.160**	**BB**	**0.0545**	**8210.81641**	**1976.72363**	**99.89956**		3	**10.084**	**BB**	**0.0605**	**7616.92383**	**1759.73987**	**99.93954**
4	11.114	BV	0.0329	7.13131e-1	3.34774e-1	0.00868		4	11.941	BB	0.0893	1.98253	3.24316e-1	0.02601
5	11.176	VB	0.0833	3.74978	6.13817e-1	0.04562								
6	13.617	BB	0.0512	1.50747	4.48821e-1	0.01834								

Results from gas chromatograms of normal and perdeuterated odorants from distinct lots and sources, as indicated from those on Table 2 and Figure 1. The percentage (Area %) of the total that constitutes the main species within each preparation is shown in bold. The batch numbers of isotopologues distinct from those on Table 1 are indicated. Distinct trace impurities were present in the 1-hexanol preparation from Lluch Essence compared to that from Fluka Analytical (Table 2). The deuterated 1-hexanol contained fewer contaminants, which, however, constituted a slightly larger fraction of the sample, since pure d13-1-HEL exhibited 99.5% purity in this sample compared to 99.7% in the one in Table 2. Similarly, h-BNZ contained fewer contaminants than that in Table 2, but it constituted a lower percentage of the sample (94.4% vs 98.7% in Table 2), because a larger proportion was benzoic acid (Ret time, 19.153). d6-BNZ also contained additional contaminants from that in Table 2 (Ret times, 8.105, 8.937) and contained a larger percentage (5.03%) of deuterated benzoic acid than the batch in Table 2. Nevertheless, the EAG responses of the two sets of batches were similar. In contrast, the normal ACP sample was purer than the original one (Table 4), and the same was true for the d8-ACP sample. Based on retention times, there are no common contaminants in the two isotopologue preparations.

**Table 4:** Amplitude differences of odorant isotopologues from distinct batches and sources

Odorant (from Table 3)	Mean amplitude	*n*	Δ Amplitude	*p* (*t* test)
h-1-HEL (Lluch Essence)	11.9367 ± 0.6336	7	0.5841 ± 0.094	<8 × 10^−4^
d13-1-HEL (X421P8)	11.3525 ± 0.6546			
h-BNZ (Fluka Analytical)	5.6187 ± 0.1380	8	−0.6275 ± 0.08	<1 × 10^−4^
d6-BNZ (X261P20)	6.2462 ± 0.1642			
h-ACP (Puriss grade, Fluka Analytical)	6.0601 ± 0.4386	10	0.3103 ± 0.062	<7.1 × 10^−4^
d8-ACP (G466P32)	5.7498 ± 0.4073			

Mean amplitudes ± SEMs of HEL, BNZ, and ACP isotopologue pairs distinct from those in Figures 1 and 2 derived from the indicated (*n*) animals are shown. Isotopologue pairs were used at the 10^−2^ dilution. The probability that the mean amplitudes evoked by the two isotopologues are significantly different is shown per odorant.

Significantly, the main measurable electrophysiological difference at the level of total antennal activity in response to odorant isotopologues is the amplitude of the EAG responses. As expected, different odorants yielded EAG traces with different overall shapes, which, however, were similar if not identical for isotopologues of the same odorant (Fig. 1A,F,K). In fact, EAGs in response to BNZ plus HEN mixtures can be differentiated from those of either odorant alone in our preparation (data not shown), which is in accord with our contention. It follows then that despite the limited resolution, if distinct ORs were activated by one isotopologue of a pair, the shapes of the resultant EAG curves would be predicted to diverge in a manner akin to the curves produced by different odorants. Instead, our results are consistent with differential activation of the same ORs, or engagement of perhaps overlapping yet distinct OR sets by each isotopologue.

### The degree of deuteration does not affect the isotopologue-specific EAG amplitude

A previous article (Gane et al., 2013) suggested that the number of deuteriums and vibrational modes seems to be important in humans, since subjects discriminated cyclopentadecanone (28 C–H/C–Ds) isotopologues, but not the much smaller acetophenone isotopologues (8 C–H/C–Ds). Therefore, we investigated whether the degree of deuteration may correlate with the differential isotopologue-specific EAG amplitudes in *Drosophila*. This is of particular interest because of reports suggesting that deuteration increases polarity in proportion with the number of deuteria and the number of heteroatoms (Wade, 1999). This decrease in hydrophobicity is apparent in the slight, but consistently faster, GC elution time of deuterated isotopologues ([Table T2][Table T3][Table T5]). Therefore, we reasoned that if the number of deuteriums per se, or the changes in polarity due to their presence, were important for differentiation, then partially deuterated odorants should evoke distinct responses from perdeuterated ones. Thus, d2-hexanol would not be readily differentiated from its normal counterpart, or at least not as well as the perdeuterated (d13-hexanol) isotopologue. In addition to hexanol, we also used isotopologues of the aromatic ketone ACP, chemically distinct from hexanol, which, in addition to the aromatic ring, contains a polar ketone functional group. The purities of ACP isotopologues are reported in Table 5.

**Table 5: T5:** Purity of normal and deuterated isotopologues of additional odorants

h-Acetophenone (Fluka analytical)							d3-Acetophenone						
Peak #	Ret time (min)	Type	Width (min)	Area (pA*s)	Height (pA)	Area %	Peak #	Ret time (min)	Type	Width (min)	Area (pA*s)	Height (pA)	Area %
1	8.634	VV	0.0532	1.75445	5.22483e-1	0.02044	1	9.212	BB	0.0459	6.59789e-1	2.27296e-1	0.00659
2	9.091	VB	0.0379	1.11003	4.33768e-1	0.01293	2	10.060	BB	0.0432	9.67617	3.62314	0.09659
3	9.499	BB	0.0409	1.46442	5.19445e-1	0.01706	3	10.384	BB	0.0443	9.54238	3.44315	0.09526
4	10.206	VB	0.0451	9.35422e-1	3.11201e-1	0.01090	4	**10.844**	**BV**	**0.0763**	**9988.40723**	**1933.71594**	**99.71190**
5	**10.909**	**VV**	**0.0605**	**8552.97363**	**1899.87683**	**99.66466**	5	11.217	VB	0.0400	7.89751e-1	3.28895e-1	0.00788
6	11.145	VV	0.0351	2.05978	8.85598e-1	0.02400	6	11.785	BB	0.0682	1.58633	5.11016e-1	0.01584
7	11.272	VV	0.0302	2.00964	1.05744	0.02342	7	12.807	BB	0.0334	5.71362	3.11383	0.05704
8	11.337	VV	0.0255	1.28859	7.71482e-1	0.01502	8	13.684	BB	0.0369	8.92039e-1	4.17685e-1	0.00891
9	11.391	VB	0.0300	1.06645	5.65312e-1	0.01243							
10	11.826	BB	0.0296	7.87661	4.26206	0.09178							
11	12.824	BB	0.0267	3.46171	1.94807	0.04034							
12	12.967	BV	0.0318	7.98368e-1	3.91961e-1	0.00930							
13	13.008	VB	0.0279	6.91700e-1	3.68989e-1	0.00806							
14	14.378	BB	0.0265	4.26080	2.42068	0.04965							
d5-Acetophenone							d8-Acetophenone (lot G466P29)						
Peak #	Ret time (min)	Type	Width (min)	Area (pA*s)	Height (pA)	Area %	Peak #	Ret time (min)	Type	Width (min)	Area (pA*s)	Height (pA)	Area %
1	**10.866**	**BB**	**0.0933**	**9092.35840**	**1730.31287**	**99.98919**	1	8.259	BB	0.0367	1.77587	8.40201e-1	0.01898
2	11.796	BB	0.0357	9.82952e-1	4.83287e-1	0.01081	2	8.850	BB	0.0328	1.34282	6.81408e-1	0.01435
							3	**10.820**	**BB**	**0.0933**	**9347.32031**	**1778.28320**	**99.90145**
							4	11.766	BB	0.0379	1.43155	6.45780e-1	0.01530
							5	12.484	BB	0.0379	2.74861	1.23874	0.02938
							6	13.712	BB	0.0391	1.48917	6.42145e-1	0.01592
							7	16.677	BB	0.0313	4.32479e-1	2.30103e-1	0.00462
h-Ethanol							d6-Ethanol						
Peak #	Ret time (min)	Type	Width (min)	Area (pA*s)	Height (pA)	Area %	Peak #	Ret time (min)	Type	Width (min)	Area (pA*s)	Height (pA)	Area %
1	**2.418**	**BB S**	**0.0205**	**4076.57593**	**3284.19873**	**1.000e2**	1	**2.387**	**BB**	**0.0360**	**4830.66064**	**2824.42505**	**1.000e2**
h-1-Pentanol							d11-1-Pentanol						
Peak #	Ret time (min)	Type	Width (min)	Area (pA*s)	Height (pA)	Area %	Peak #	Ret time (min)	Type	Width (min)	Area (pA*s)	Height (pA)	Area %
1	2.711	BB	0.0231	6.06239e-1	4.13028e-1	0.00853	1	3.443	BB	0.0421	11.47077	6.04865	0.15594
2	4.421	BB	0.0363	1.78695	7.93818e-1	0.02515	2	3.984	BB	0.0381	24.50063	12.94281	0.33307
3	**5.023**	**BB**	**0.0618**	**7100.83350**	**1662.52856**	**99.94445**	3	**4.528**	**BB**	**0.0792**	**7319.98291**	**1443.77832**	**99.51099**
4	5.750	BB	0.0231	1.55354	1.05958	0.02187							
h-1-Octanol							d17-1-octanol						
Peak #	Ret time (min)	Type	Width (min)	Area (pA*s)	Height (pA)	Area %	Peak #	Ret time (min)	Type	Width (min)	Area (pA*s)	Height (pA)	Area %
1	5.607	BB	0.0376	8.33811	4.49661	0.10979	1	5.692	BB	0.0379	23.70400	12.64659	0.25730
2	6.696	BB	0.0325	2.99909	1.70881	0.03949	2	7.063	BB	0.0385	11.61009	6.02909	0.12603
3	8.018	BV	0.0456	1.06406	4.85292e-1	0.01401	3	**8.644**	**BB**	**0.1126**	**9162.00488**	**1206.64636**	**99.45199**
4	8.165	VB	0.0400	2.65737	1.29295	0.03499	4	9.924	BB	0.0483	1.24629	5.13031e-1	0.01353
5	8.821	BB	0.0560	27.26840	7.94571	0.35904	5	10.233	BB	0.0438	4.07517	1.99705	0.04424
6	**9.302**	**BB**	**0.1036**	**7552.50098**	**1054.55908**	**99.44269**	6	11.328	BB	0.0524	4.05706	1.45943	0.04404
							7	13.140	BB	0.0522	2.07261	7.51371e-1	0.02250
							8	13.614	BB	0.0537	1.37058	4.24300e-2	0.01488
							9	14.565	BB	0.0490	2.34988	9.43809e-1	0.02551

Results from gas chromatograms of normal, partially deuterated, and perdeuterated additional odorants used herein. The percentage (Area %) of the total that constitutes the main species within each preparation is shown in bold. For acetophenone isotopologues, the purity of all preparations was >99.7%. The contribution of all contaminants was negligible as it ranged below 0.1%. Both ethanol isotopologues are 100% pure. For pentanol, the normal odorant was nearly pure (99.9%), while the deuterated isotopologue was highly pure at 99.5%, with two main, albeit low-level, contaminants: peak 1 at 0.16% and peak 2 at 0.33%. For octanol, the normal isotopologue was highly pure (99.4%), while the deuterated isotopologue is equally pure (99.5%). Although greater in number, the contribution of contaminants in the deuterated isotopologue is minor, except for the peak at 5.692 contributing 0.26% to the total. This peak is shared with the normal isotopologue at 5.607 and contributes 11% of the total while the major contaminating peak at 8.821 contributes 0.36%.

As is apparent by the representative traces in Figure 2A, normal hexanol yielded significantly higher EAG amplitudes than those elicited by all of its deuterated isotopologues, and this is reflected in the data from partially deuterated versus normal isotopologue pairs in Figure 2B–D. This surprising result was independently verified by challenging individual flies with the di-deuterated versus perdeuterated hexanol, which yielded strikingly similar traces, which are illustrated in Fig. 2E and are detailed in Fig. 2F, underscoring the lack of significant EAG differences. The calculation of Δ amplitude for each deuterated and normal isotopologue revealed that it was not significantly different across the range of hexanol deuteration tested (Fig. 2G). Similar responses were obtained over a range of dilutions of d2 and d13-HEL (Table 6). The mean amplitudes were proportional to the dilutions of the odorants, as expected. Importantly, the response amplitudes generated by each of the isotopologues at a given dilution were not significantly different from each other, as the *p* values and the near zero Δ amplitude values indicate (Table 6). It should be noted that the results from the 10^−2^ HEL isotopologue dilution are independent of and confirm those reported in Figure 2, F and G.

**Table 6:** Lack of differences in the amplitudes of partially and perdeuterated odorant pairs over a range of dilutions

Odorant	Mean amplitude	*N*	Mean Δ amplitude	*p* (*t* test)
d2-1-HEL 1 × 10^−4^	4.0675 ± 0.1789	7	−0.0392 ± 0.009	0.3213
d13-1-HEL 1 × 10^−4^	4.1068 ± 0.1697			
d2-1-HEL 1 × 10^−3^	6.4915 ± 0.1603	7	0.1438 ± 0.129	0.3085
d13-1-HEL 1 × 10^−3^	6.3477 ± 0.1641			
d2-1-HEL 1 × 10^−2^	12.2325 ± 0.4389	7	−0.092 ± 0.195	0.653
d13-1-HEL 1 × 10^−2^	12.3246 ± 0.3804			
d3-ACP 1 × 10^−4^	3.4181 ± 0.2490	7	0.0437 ± 0.054	0.4490
d8-ACP 1 × 10^−4^	3.3743 ± 0.2554			
d3-ACP 1 × 10^−3^	4.6298 ± 0.2093	10	−0.1004 ± 0.064	0.1542
d8-ACP 1 × 10^−3^	4.7302 ± 0.2235			
d3-ACP 1 × 10^−2^	6.2129 ± 0.6041	7	0.0447 ± 0.133	0.7478
d8-ACP 1 × 10^−2^	6.1682 ± 0.5080			

The dilutions and the resultant mean amplitudes ± SEMs of perdeuterated and minimally deuterated HEL and ACP isotopologue pairs collected from the indicated (*n*) number of animals are shown. The resultant mean Δ amplitudes ± SEMs are also shown, as well as the probabilities from paired *t* tests (*p* value, *t* test) that the mean responses to each pair of isotopologues at each dilution are significantly different. Significant differences were not uncovered.

Similar results were obtained with acetophenone isotopologues (Fig. 2H–N), where again the normal ACP yielded significantly larger EAG amplitudes than any of the deuterated odorants (Fig. 2Ι–K). Importantly, the Δ amplitude values between normal and trideuterated, pentadeuterated, or perdeuterated odorants were statistically indistinguishable (Fig. 2N), a conclusion confirmed independently by the identical EAGs elicited when individual animals were challenged with d3- and d8-acetophenone (Fig. 2L,M). Again, the lack of difference in amplitudes yielded by the d3 and d8 isotopologues held over a range of dilutions (Table 6) and confirmed the results mentioned above.

Therefore, the lack of EAG amplitude differences between perdeuterated and partially deuterated isotopologues of HEL and ACP is not a consequence of saturating odorant at the 10^−2^ dilution. It follows then that the incorporation of as few as two deuteria is sufficient to evoke measurable differential EAG amplitudes from their normal isotopologues. Moreover, the effect of multiple C–D bonds in a molecule does not appear to be linear.

The reduced volatility of the deuterated odorants does not explain the EAG amplitude differences because, if it were responsible, isotopologues with fewer deuteria (i.e., d2-hexanol and d3-acetophenone) would be expected to elicit EAG amplitudes that were similar or identical to their normal counterparts. Furthermore, differences cannot be attributed to impurities, because the hexanol isotopologues are highly pure (Tables 2, 3), and impurities vary in the preparations in a fashion that is discordant with the amplitude differences reported here. In agreement, independent lots of ACP isotopologues at the 10^−2^ dilution (Table 3) yielded results (Table 4) similar to those in Figure 2K.

**Figure 3.**
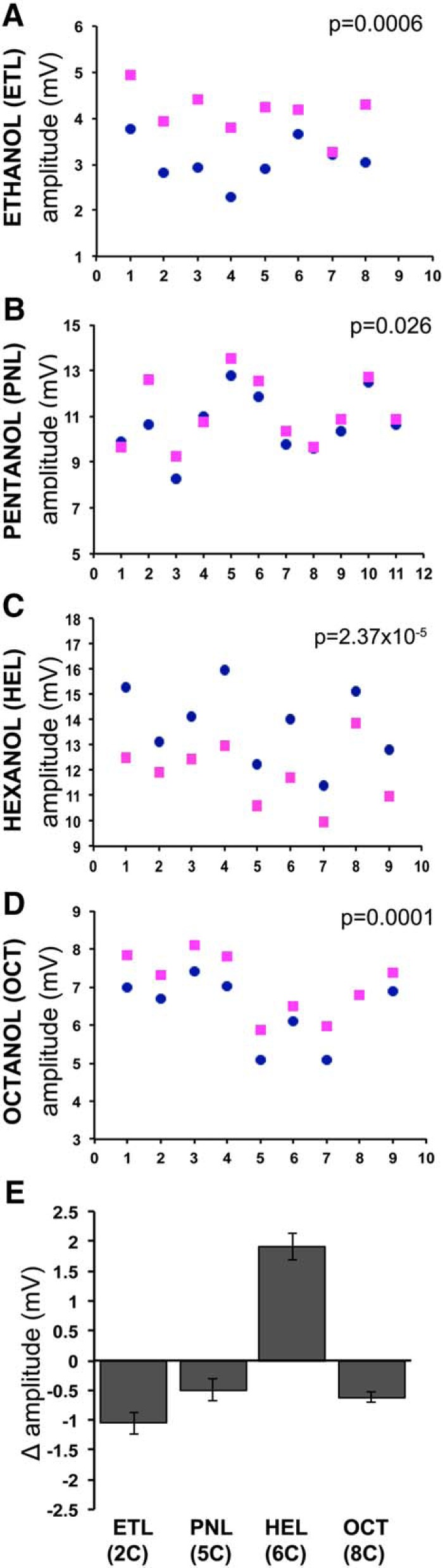
Isotopologue-specific EAG amplitudes of normal and deuterated alcohols. ***A–D***, The maximal amplitudes for each fly tested with the normal (dark blue dots) or deuterated (magenta squares) isotopologue of the 2-carbon ethanol and d6-ethanol (ETL), the 5-carbon pentanol and d11-pentanol (PNL), the 6-carbon hexanol and d13-HEL, and the 8-carbon octanol and d17-octanol (OCT) at the 10^−2^ dilution. The number of flies tested with each isotopologue is shown in the abscissas, with each pair of blue and magenta dots representing the responses per single fly, while the ordinate scales have been adjusted for maximal resolution. The significance of the uncovered differences after paired *t* tests is shown on the graphs. The data in ***C*** are the same as in Figure 1*D*. ***E***, Quantification of the collective differential response per odorant isotopologue shown as Δ amplitude ± SEM. The data for the hexanol isotopologues are the same as in Figure 1. The actual Δ amplitude values and their SEMs are summarized as follows:

In the case of hexanol and acetophenone isotopologues, the number of deuteria varied, but the size of the molecule that carried them was constant. We aimed to independently verify these surprising results by asking whether the size of the deuterated molecule is important for the differential EAG amplitudes. To keep the functional group unaltered and stereochemistry as similar as possible, we selected four alcohols, including 1-hexanol, to ask whether size may be a relevant differentiating parameter, and tested normal and deuterated pairs at the 10^−2^ dilution. With the exception of 1-pentanol, where the difference was marginal (Fig. 3B), ethanol and octanol isotopologues evoked highly significant EAG differences (Fig. 3A,D). However, unlike the response to hexanol isotopologues (Fig. 3C), where the normal odorant elicited larger EAG amplitudes, deuterated ethanol, pentanol, and octanol evoked larger responses (Fig. 3E). This difference does not appear to depend on the size of the molecule since hexanol is in the middle of the size range, or on the number of deuteria it carries (13), which is also in the middle of the range (6–17 deuteria) examined.

**Table:** 

Odorant	Mean h-	SEM	Mean d-	SEM
ETL	3.0746	0.1671	4.1313	0.1733
PNL	10.6673	0.3975	11.1639	0.4391
HEL	13.7872	0.5066	11.8815	0.4079
OCT	6.4527	0.2811	7.0699	0.2752

Because EAGs report total OR activity, the response summarizes the activation and inhibition of ORs responsive to a particular isotopologue. Maximally activated ORs are likely to contribute the majority of the activity reported in EAG amplitudes. It follows then that the isotopologue-specific EAG amplitudes reflect differences in the subsets of maximally activated ORs. The direction of the Δ amplitude difference between hexanol and the other alcohols may be consequent of the number of ORs activated or inhibited by each odorant isotopologue. Hence, within the subset of hexanol-activated ORs, the majority yield maximal activities with the normal odorant, and fewer with d13-HEL. The converse could be the case for ORs activated by ethanol pentanol and octanol, as suggested by their Δ amplitudes (Fig. 3E). Alternatively, particular ORs respond only to one of the two isotopologues, and, if so, more appear to be responsive to normal odorants than d13-HEL, with the converse for ethanol, pentanol, and octanol.

Interestingly, the Δ amplitude ranges are similar for ethanol with 6 deuteria and octanol with 17 deuteria (Fig. 3E). Therefore, as suggested above, increasing the number of deuteria does not proportionally increase the EAG difference from that elicited by the normal odorant. Moreover, inasmuch as odorant size determines volatility, it does not predict which isotopologue will evoke the larger EAG, as exemplified by the opposite Δ amplitude effects for pentanol and hexanol. Therefore, the differential responses to isotopologues appear to depend primarily on the presence of deuterium and to be independent of its actual numbers per molecule. Collectively, the results do not support the hypothesis that deuteration-mediated polarity changes are responsible, or contribute to the differential EAG amplitudes evoked by deuterated versus normal odorants. In contrast, the results are consistent with the notion that ORs are sensitive to the characteristic vibrational frequency of at least two, and perhaps a single C–D, bond to differentiate deuterated odorants from their normal isotopologues in *Drosophila*.

### Perireceptor effects are not major contributors to isotopologue differentiation

Perireceptor mechanisms that potentially interplay or interfere with direct odorant engagement by the ORs have recently been reported (Heydel et al., 2013) and could in principle affect differential isotopologue perception. The OBPs bind odorants with high affinity, probably based on their overall shape or functional groups, and act as carriers to the ORs (Swarup et al., 2011; Leal, 2013). Although isotopologues do not differ in shape, EAG amplitude differences may reflect differential diffusion or transport through the sensillar lymph of the slightly heavier and more polar deuterated isotopologues to the ORs.

**Figure 4.**
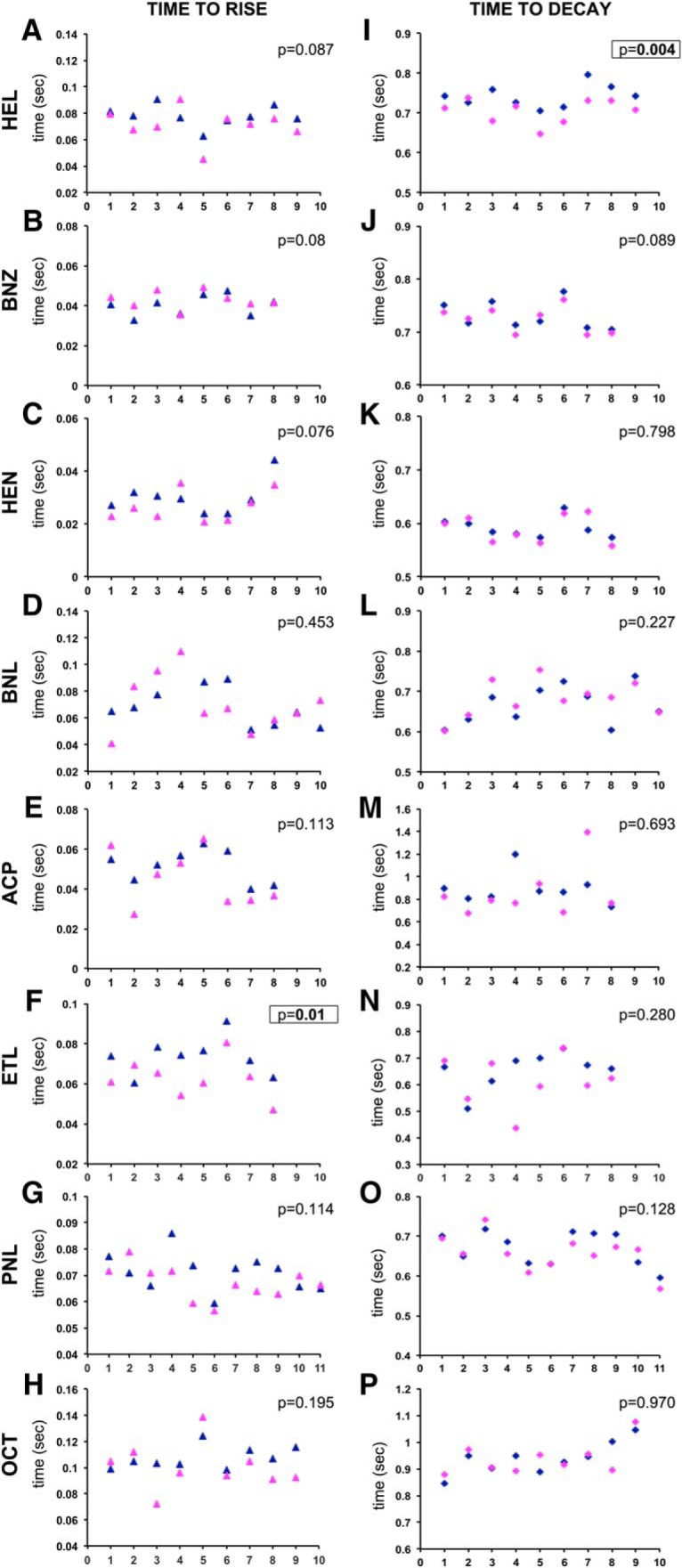
Isotopologues elicit similar EAG activation and decay rates. ***A–P***, The times required to achieve two-thirds of the maximal amplitude (time to rise, ***A–H***) and times required to recover to one-third of the maximal amplitude (time to decay, ***I–P***) are shown for isotopologues of all odorants used in this study. Dark blue and magenta triangles are used for the rise times due to normal and deuterated isotopologues, respectively, and conversely dark blue and magenta diamonds are used for decay times. The number of flies tested with each isotopologue is shown in the abscissas, and the ordinate scales have been adjusted for maximal resolution. The probability that paired *t* tests uncovered isotopologue-specific differences in rise and decay times is shown, with significant differences in bold and boxed. ETL, Ethanol; PNL, 1-pentanol; OCT, 1-octanol.

To investigate whether isotopologues are transported differentially to the respective ORs, we estimated the time required to reach two-thirds of the maximal EAG amplitude (rise time) upon odorant exposure (Alcorta, 1991). The results presented in Figure 4A–H did not reveal significant differences (paired *t* tests) in rise time for all isotopologues tested except for ethanol, where rise times in response to the deuterated odorant were found significantly slower in seven of the nine animals tested (Fig. 4F). Because the deuterated isotopologue evoked a slower response, it may reflect less efficient OBP engagement or transport to the cognate OR because of its increased polarity. However, this is unlikely to be generalized and underlie the higher EAG amplitudes evoked by deuterated pentanol and octanol (Fig. 3E), because their rise times were not significantly different from those elicited by their normal counterparts (Fig. 4G,H). Moreover, rise time differences were not seen for hexanol, where the normal isotopologue evokes higher EAG amplitudes (Fig. 3E). Similar data were obtained with higher isotopologue dilutions (data not shown). Collectively, isotopologue-specific rise time differences were not observed in seven of the eight odorant pairs tested, and ethanol may be an exception because of its size. Therefore, our data do not support the notion that the isotopologue-specific EAG amplitude differences we uncovered (Figs. 1–3) reflect differential diffusion or OBP-dependent transport (Heydel et al., 2013; Leal, 2013) to the ORs.

Furthermore, we determined the time required for the response to decay to one-third of the maximum (fall time) as a measure of OR activity after stimulus removal. We reasoned that the slightly heavier deuterated isotopologues may require more time to be cleared from the respective ORs, evoking longer OR activities, perhaps underlying the differential EAG amplitude. Overall, the data (Fig. 4I–P), do not reveal isotopologue-specific significant differences in fall time for all odorants tested except for hexanol (Fig. 4I), which is also illustrated by the difference in the recovery (right side) portion of the average traces at 10^−2^ and 10^−3^ in Figure 1A. This was also observed with the alternative HEL isotopologues in Table 6 (data not shown). However, the longer time required to return to baseline upon d13-HEL exposure does not correlate with the differential amplitudes, as it is the normal isotopologue that evokes the higher EAG (Fig. 1A–E). Collectively, the data indicate that, in general, OR engagement by the slightly heavier deuterated odorants and residual OR activity is not differentially affected by isotopologues so as to underlie the observed EAG amplitude differences.

Biotransformation and detoxification enzymes contribute to odorant inactivation in the perireceptor space (Martin et al., 2013). The main such enzymes are of the cytochrome P450 family in the *Drosophila* antenna (Wang et al., 1999; Brandt et al., 2002). If biotransformation is required before OR engagement for the odorants used in this study, deuterated odorants could be processed at a different rate from their normal isotopologues, as described previously (Swiderek and Paneth, 2013). Differences in processing rates could result in submaximal OR activation, which could account for the observed amplitude effects. Alternatively, P450 activity may be necessary to clear the odorant from the perireceptor space and deuterated odorants may in fact be cleared at a lower rate yielding differential EAG properties and amplitudes. Therefore, we sought to inhibit the P450 family and determine the effects on the EAGs of selected isotopologue pairs.

**Figure 5.**
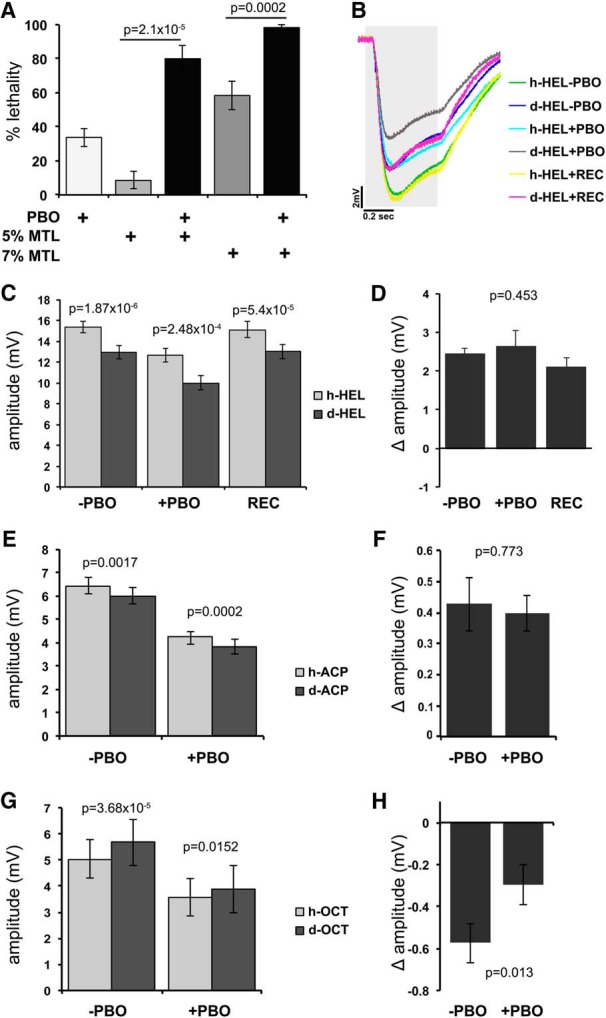
P450 inhibition does not alter the isotopologue-specific differential responses. ***A***, Flies were exposed to PBO dissolved in acetone. The mean lethality ± SEM due to exposure to 0.25% (v/v) PBO in acetone alone (PBO), 5% and 7% methanol (MTL) alone (v/v in minimal food), and combinations thereof is shown (*n* = 8 for each). PBO significantly (*t* test-derived probabilities shown above the bars compared as indicated) augmented the lethality precipitated by either 5% or 7% methanol, demonstrating that under the conditions of the experiment it actually inhibits *Drosophila* P450s. ***B***, Flies were exposed to PBO or the acetone vehicle (−PBO) for 18 h at 25°C. Another group of animals was exposed to PBO for 18 h and then allowed to recover on minimal food for another 18 h (REC). Average EAG traces from treated (+PBO), control (−PBO), and REC flies exposed to normal (h-HEL) and d13-hexanol (d-HEL) isotopologues. The gray area indicates the timing and duration of stimulation. ***C***, The mean EAG amplitudes ± SEM of treated, untreated, and recovered animals demonstrated that the differential responses to HEL isotopologues remain significant (probabilities from paired *t* tests are shown above the relevant bars) despite the PBO treatment. ***D***, Δ amplitudes calculated from the data in ***C***. ANOVA (F_(2,23)_ = 0.819) did not indicate significant differences (*p* = 0.453) among groups, indicating that the isotopologue-specific differences remain despite the PBO treatment. ***E***, Mean EAG amplitudes ± SEM of PBO and vehicle-treated (−PBO) animals exposed to normal and d8-ACP show that the isotopologue-specific responses remain significant (paired *t* test probabilities above the respective bars) despite PBO treatment. ***F***, Δ Amplitudes calculated from the data in ***E***. The isotopologue-specific differences remain despite PBO treatment as ANOVA (*F*_(1,14)_ = 0.087) did not indicate significant differences (*p* = 0.773) among groups. ***G***, Mean EAG amplitudes ± SEM of PBO and −PBO animals exposed to normal and d17-octanol (OCT) indicate a marginal (*p* = 0.0152, paired *t* tests) isotopologue-specific response after PBO treatment. ***H***, Δ Amplitudes calculated from the data in ***G***. As indicated in ***G***, the isotopologue-specific differences are decreased upon PBO treatment as ANOVA (*F*_(1,16)_ = 7.727) indicated a significant difference (*p* = 0.013) among groups.

CYPs were inhibited with PBO, using the protocol of Wang et al. (2013). Initially, we verified that PBO exposure enhanced methanol toxicity as reported (Wang et al., 2013). Then the lowest effective PBO concentration that enhances methanol toxicity without being excessively toxic itself was determined as 0.25% (v/v). If P450s were essential for isotopologue differentiation, their inhibition should eliminate the isotopologue-specific amplitude differences. Control EAG traces upon stimulation with hexanol isotopologues yielded differences comparable with those described and quantified in Figure 1 (Fig. 5B, compare green trace, dark blue trace). Flies exposed to PBO for 18 h responded to hexanol isotopologues differentially (Fig. 5B, compare light blue trace, dark gray trace), albeit with reduced EAG amplitudes (Fig. 5C). This suggests that PBO inhibits the enzymes necessary for the activation of all ORs contributing to the differential maximal EAG amplitudes, or that the reduced response is due to compound toxicity on the ORs. However, although the EAG amplitudes were reduced, the isotopologue-specific differential effect remained similar to that in untreated animals (Fig. 5D). Moreover, PBO treatment did not differentially affect the rise and fall times in response to hexanol isotopologues (data not shown), suggesting that odorant transport and OR engagement were not affected.

To determine whether the reduced amplitude was caused by OR depression or attrition due to PBO toxicity, we allowed the flies to recover for 18 h following PBO treatment. Surprisingly, recovery resulted in EAG amplitudes that were virtually indistinguishable from those of controls (Fig. 5B, compare yellow traces, green traces, and dark blue traces, magenta traces; Fig. 5C). Again the isotopologue-specific amplitude differences in all three groups were not significantly different (Fig. 5D). Rise and fall times in flies recovered from PBO treatment were also similar to those from controls (data not shown). Therefore, PBO treatment is not irreversibly toxic to ORs, and P450 family enzymes contribute to maximal hexanol detection, but their activity does not affect isotopologue differentiation. Similar results were obtained with the unrelated aromatic ACP isotopologues (Fig. 5E,F), which is indicative of the generality of the above conclusions. We also tested 1-octanol, where the deuterated odorant elicited larger EAGs. In this situation, PBO treatment did not abolish (Fig. 5G), but reduced the isotopologue differences such that they became marginally significant (Fig. 5H). This appears to result from reduced PBO-mediated inhibition of the amplitude elicited by h-1-OCT relative to that from its deuterated isotopologue, as suggested by their Δ amplitude. Therefore, P450s could be important for perireceptor processing or clearance of normal 1-octanol, but this is a likely exception and alone does not account for the specific EAG differences of other isotopologue pairs.

Collectively our data do not lend support to the notion that, in general, isotopologues are engaged or processed differentially enough by the *Drosophila* antenna to correlate with the observed significant differences in EAG amplitudes. This agrees with kinetic analyses indicating that P450s do not degrade deuterated substrates faster than their normal counterparts (Guengerich, 2013).

### Evolutionary conservation of the differential isotopologue response

Isotopologue discrimination salient to behavioral choices has been described recently for *Drosophila* and bees (Franco et al., 2011; Bittner et al., 2012; Gronenberg et al., 2014). Isotopologue discrimination limited to a single or a few odorants has also been reported for the flour beetle *Tribolium castaneum*, the American cockroach *Periplaneta americana* (Meloan et al., 1988), and more recently in humans for a macrocyclic musk (Gane et al., 2013), indicating broad evolutionary conservation. Because *Drosophila* ORs are highly diverse and fast evolving (de Bruyne et al., 2010), we wondered whether the specific responses to isotopologues described herein for *D. melanogaster* would be conserved within the genus. Hence, we used EAGs to ask whether representative species within the genus respond similarly to isotopologues. Toward that end, we used hexanol, benzaldehyde, hexanone, and benzonitrile isotopologues to obtain EAGs from the equivalent location as that for *D. melanogaster* antennae in the sibling species *Drosophila simulans*, the more distant *D. pseudoobscura*, and *D. virilis*, which is separated from *D. melanogaster* by >40 million years (de Bruyne et al., 2010).

**Figure 6.**
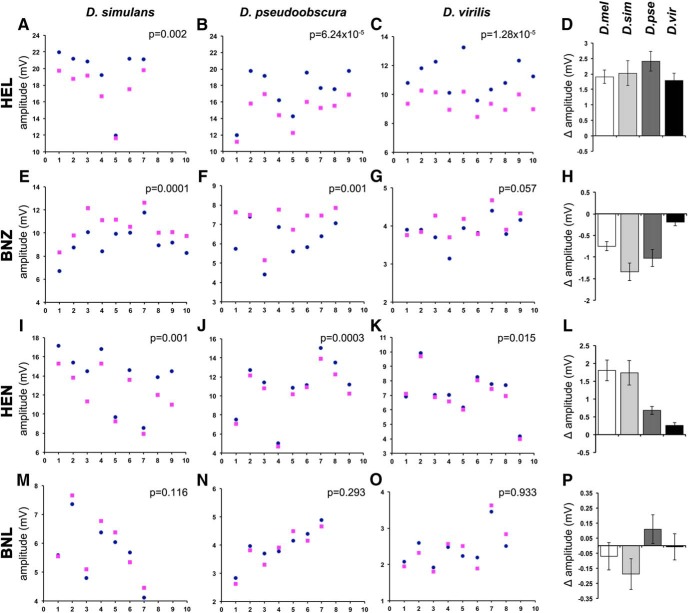
The differential response to isotopologues is conserved within the genus *Drosophila*. ***A–C***, ***E–G***, ***I–K***, ***M–O***, Raw EAG amplitudes from the antennae of *D. simulans* (D.sim), *D. pseudoobscura* (D.pse), and *D. virilis* (D.vir) in response to hexanol (***A–C***), benzaldehyde (***E–G***), hexanone (***I–K***), and benzonitrile (***M–O***) isotopologues (dark blue dots for the normal and magenta squares for the deuterated odorant). The ordinate scales have been adjusted to allow maximal resolution. The probability (paired *t* tests) that isotopologue-specific differences are uncovered is indicated on the panels. Δ Amplitudes calculated from the data in the previous panels are shown in ***D*** for hexanol, in ***H*** for benzaldehyde, in ***L*** for hexanone, and in ***P*** for benzonitrile. The relevant Δ amplitudes for *D. melanogaster* from Figure 1 are added for comparison. ANOVA did not indicate (*F*_(3,34)_ = 0.992, *p* = 0.409) significant differences in Δ amplitudes for HEL (***D***) or BNL (*F*_(3,28)_ = 1.518, *p* = 0.232; ***P***). However, ANOVA indicated significant differences in Δ amplitudes for BNZ isopotologues (*F*_(3,31)_ = 9.672, *p* < 3.25 × 10^−5^), which subsequent Tukey’s HSD test indicated were due to differences in the Δ amplitude values for *D. simulans* and *D. pseudoobscura* compared with that from *D. virilis* (α = 0.05). Similarly, ANOVA indicated differences (*F*_(3,31)_ =10.802, *p* = 0.232) in Δ amplitudes elicited by HEN exposure (***L***). Tukey’s HSD test revealed that the Δ amplitude values of *D. melanogaster* and *D. simulans* were significantly different from those of *D. virilis* (α = 0.05).

All species responded differentially to hexanol isotopologues (Fig. 6A–C), and, importantly, as for *D. melanogaster* (Fig. 1D), the normal odorant evoked significantly higher amplitudes than d13-HEL (Fig. 6D). Furthermore, as for *D. melanogaster* (Fig. 1I), d6-benzaldehyde evoked significantly higher amplitudes than its normal counterpart in *D. simulans* and *D. pseudoobscura* (Fig. 6E,F,H). However, d6-BNZ evoked marginally larger amplitudes in the distant *D. virilis* (Fig. 6G,H). A similar pattern was uncovered with 2-hexanone isotopologues, where *D. melanogaster* (Fig. 1N), *D. simulans*, and *D. pseudoobscura* responded with significantly larger EAG amplitudes to the normal than the deuterated odorant (Fig. 6J,K), but again the difference was marginal for *D. virilis* (Fig. 6K). Importantly, *D. simulans*, *D. pseudoobscura*, and *D. virilis* presented highly variable and not significantly different responses to BNL isotopologues (Fig. 6M–O). Therefore, similar to *D. melanogaster* these species spanning the breath of the genus, do not differentiate benzonitrile isotopologues, indicating that the mechanism of isotopologue differentiation is conserved and involves the detection of molecular vibrations.

In summary, although to different degrees in accord with species occupying diverse niches and separated by significant evolutionary distance, members of the genus *Drosophila* presented conserved differential EAG amplitudes to odorant isotopologues. Significant differences in the magnitude of the differential response among the species were detected for benzaldehyde (Fig. 6B) and hexanone (Fig. 6C), and this is not totally unexpected considering the evolutionary distance and the high variability of ORs. Importantly, however, the isotopologue from each pair eliciting the higher EAG response was conserved and so was the inability to distinguish the benzonitrile isotopologues. This likely reflects isotopologue-specific activities of OR subsets for sibling and distant species, and suggests that, as for *D. melanogaster* (Franco et al., 2011; Bittner et al., 2012), isotopologues may elicit differential behavioral responses.

We selected hexanol isotopologues to address this question, because they elicited equivalent responses in terms of Δ amplitude (Fig. 6D). We simplified the Pavlovian conditioning assay used previously (Franco et al., 2011), reasoning that the isotopologue in whose presence animals are punished should be selectively avoided, even in the absence of an unpunished odorant. In contrast, if not differentiated, both isotopologues should elicit identical responses. Comparative pilot experiments indicated that this modified assay affords better resolution than Pavlovian conditioning (data not shown). Odorant concentrations (Table 1 in Materials and Methods) were adjusted in control experiments, as previously done (Franco et al., 2011), to elicit as balanced a response from naive animals as possible.

**Figure 7.**
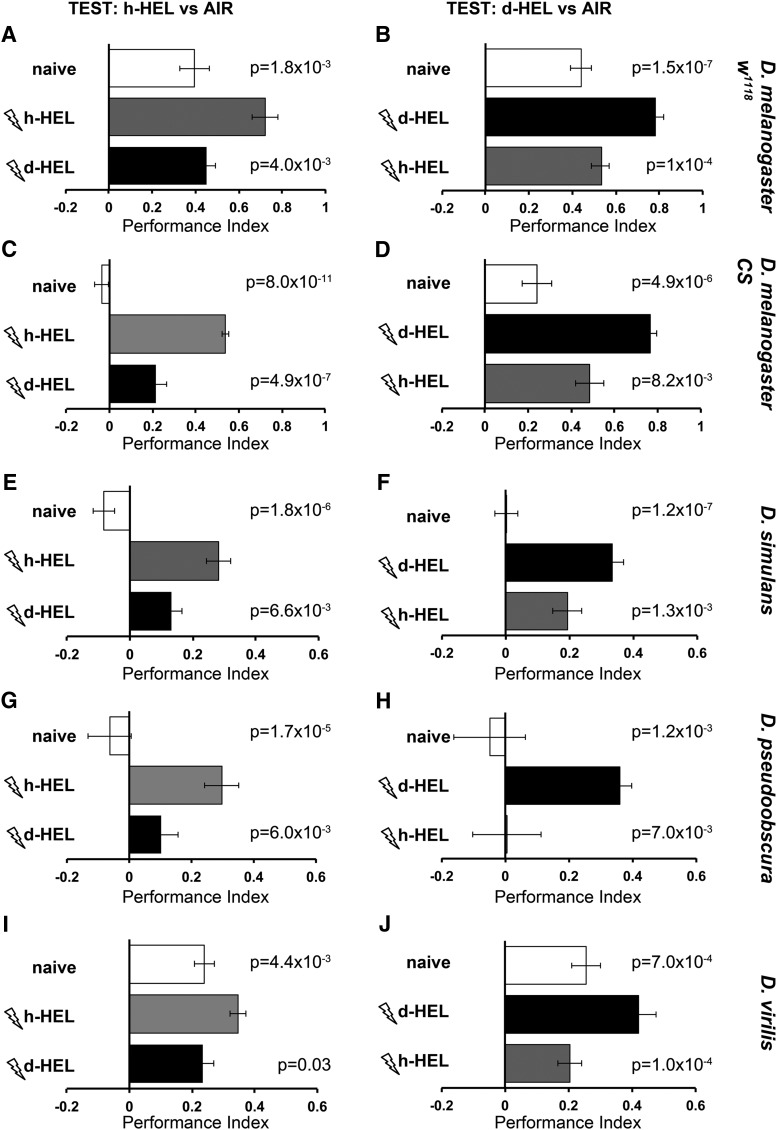
Behavioral discrimination of 1-hexanol isotopologues within the genus *Drosophila*. The mean 1-hexanol isotopologue avoidance ± SEM of complementary experiments is shown. The *Drosophila* strains and species tested are indicated on the right of each pair of experiments. The graphs are shown horizontally to reflect the actual distribution of the flies in the left and the right arms of the T-maze. Room air is shown delivered on the right arm, whereas the odorant on the left, although in actuality the side of air and odorant delivery were alternated semi-randomly. Open bars indicate the naive response to the indicated isotopologue vs room air. ***A***, ***C***, ***E***, ***G***, ***I***, Flies were exposed to 12–90 V electric footshocks (thunderbolts) in the presence of either normal (h-HEL, gray bars) or perdeuterated (d13-HEL, black bars) 1-hexanol and then tested for avoidance of the normal isotopologue vs air. The complementary experiments are shown in ***B***, ***D***, ***F***, ***H***, and ***J***, with flies exposed to electric footshocks (thunderbolts) in the presence of either normal (h-HEL, gray bars) or perdeuterated (d-HEL, black bars) 1-hexanol and then tested for avoidance of the perdeuterated odorant vs air. Differences in the performance of each group were investigated by an initial ANOVA followed by least square means contrast analysis. The group trained to avoid the same isotopologue as used for testing was denoted as the control group, and the probabilities that it performed differently than naive or animals trained to the other isotopologue are shown above each relevant bar. *n* ≥ 8 for all groups.

Naive *D. melanogaster* strain *w^1118^* flies showed mild avoidance for h-hexanol (Fig. 7A, naive), which was significantly enhanced upon prior punishment in its presence (Fig. 7A, h-HEL-trained). However, h-hexanol avoidance after punishment in the presence of d13-HEL was not enhanced and remained significantly different from the performance of animals trained with normal hexanol (Fig. 7A, d13-HEL trained). In the converse experiment, avoidance of d-hexanol by naive animals (which was similar to that of the h-isotopologue), was significantly enhanced by prior punishment in its presence (Fig. 7B, d13-HEL trained). However, punishment in the presence of the normal odorant did not result in enhanced d13-HEL avoidance (Fig. 7B, h-HEL trained). Identical results were obtained with the Canton-S strain of *D. melanogaster* (Fig. 7C,D), demonstrating the stability and reproducibility of the assay. Therefore, in accord with prior results with other odorants (Franco et al., 2011), *D. melanogaster* readily differentiate hexanol isotopologues at the behavioral level and respond accordingly.

Importantly, *D. simulans* (Fig. 7E,F), *D. pseudoobscura* (Fig. 7G,H), and *D. virilis* (Fig. 7I,J) also readily differentiated between hexanol isotopologues, albeit with lower performance indices for *D. virilis.* Therefore, flies within the genus *Drosophila* present conserved selective avoidance of isotopologues linked to electric footshocks. This indicates that these species differentiate isotopologues not only electrophysiologically at the level of the receptors, but also as salient stimuli to drive behavioral choices, which is in agreement with previous publications (Franco et al., 2011; Bittner et al., 2012; Gronenberg et al., 2014).

## Discussion

We demonstrate that a range of odorant isotopologues elicited distinct electrical changes in Drosophilid antennae, yielding isotopologue-specific EAG amplitudes. As described previously (Franco et al., 2011; Bittner et al., 2012) and herein, such differential responses are behaviorally salient, suggesting that isotopologues are evaluated and identified as distinct odors in higher brain centers, such as the lateral horn and the mushroom bodies (Galizia, 2014). The contribution of such higher brain centers to isotopologue identification driving quantifiable behavioral choices is illustrated by the reported graded behavioral responses to partially and perdeuterated ACP isotopologues (Franco et al., 2011; Bittner et al., 2012), which was not observed at the level of OR activities (Fig. 2A–G).

Our results indicate strongly that ORs differentiate odorant isotopologues based on the presence of C–D bonds. Because odorants do not bind ORs covalently, the known differential strengths of the C–H and C–D bonds (Wade, 1999; Swiderek and Paneth, 2013) are not possible mediators of isotopologue differentiation. Furthermore, the effects of deuteration on covalent interactions, such as hydrogen bonding, and ionic and van der Waals interactions, are negligible (Wade, 1999; Swiderek and Paneth, 2013), indicating that these physical properties also do not contribute to isotopologue differentiation. Conversely, deuteration is reported to increase polarity (Wade, 1999), a modification of potentially significant consequences to the hydrophobic odorant molecules. Reduced hydrophobicity could decrease the time that deuterated odorants transit the aqueous sensillar lymph, reflected as an isotopologue-specific reduction in rise time, which was not observed (Fig. 4). OBPs are the likely escort of the hydrophobic odorants through the aqueous lymph to the ORs. OBPs appear to bind specific odorants, suggesting selectivity based on structural determinants (Heydel et al., 2013; Martin et al., 2013). If so and because isotopologues are of identical structure, it is unlikely that differential OBP binding mediates isotopologue differentiation. Because deuterated odorants are slightly heavier, OBPs could escort isotopologues to ORs at different rates, which are potentially reflected in the rise times. However, with the exception of ethanol, isotopologue-specific differences in rise times were not observed (Fig. 4), suggesting that, generally, OBPs are not major contributors to isotopologue differentiation.

Furthermore, the isotopologue-specific EAG amplitude differences do not depend on antennal enzymes of the cytochrome P450 family (Fig. 5). Additional enzymes of the glutathione *S*-transferase, monoxygenase, and 5-diphosphoglucose disodium salt-glucoronosyltransferase families have been reported in the sensillar lymph (Wang et al., 1999; Martin et al., 2013), but as they mainly function in rapid odorant inactivation, they are unlikely to contribute to isotopologue differentiation. These enzymes may influence signal duration, but not its onset or amplitude, as reported for another *Drosophila* sensillar enzyme, the EST-6 esterase (Chertemps et al., 2015). Therefore, EAG amplitude-dependent differentiation is unlikely to be the consequence of enzymatic activities in the sensillar lymph, strongly suggesting that perireceptor events do not contribute significantly to isotopologue differentiation.

Therefore, our collective results, which are in agreement with previous evidence (Franco et al., 2011; Bittner et al., 2012), strongly suggest that Drosophilid ORs are sensitive to the vibrations of odorant molecules since this is the main physical property relevant to olfaction that differentiates the isotopologues.

Ιt is not unreasonable to expect that partially deuterated isotopologues would yield responses similar to those from normal odorants. However, d2-HEL versus d13-HEL and d3-ACP versus d8-ACP yielded practically superimposable traces (Fig. 2E,L) over a range of concentrations (Table 6). Moreover, rise times were nearly identical (0.078 ± 0.002 s for normal, 0.072 ± 0.003 s for d2-HEL, and 0.071 ± 0.002 s for d13-HEL; 0.052 ± 0.003 s for normal, 0.059 ± 0.001 s for d3-ACP, and 0.055 ± 0.001 for d8-ACP) at the 10^−2^ dilution. The notion that a few or a single C–D bond suffices for isotopologue differentiation is also in accord with published evidence that the C–D vibration at 2150 cm is perceived (Franco et al., 2011) as that of the C≡N functional group with which it shares vibrational frequency. This is independently supported at the OR level where the vibrational frequency of C≡N in benzonitrile overlaps that of the C–D bond in deuterated BNL. Hence, in a manner similar to partially deuterated and perdeuterated compounds, the two BNL isotopologues yield identical EAG traces (Fig. 1P–T).

Therefore, it appears that *Drosophila* ORs differentiate isotopologues on the basis of few bonds or vibrations with frequencies distinct from that of the typical C–H vibration at 3000 cm. However, typical odorants do not contain deuterium, but rather functional groups that present distinct vibrational modes and frequencies, which have been proposed to mediate odorant character (Turin and Yoshii, 2003). Thus, our observation that as few as a couple of C–D bonds suffice to differentiate isotopologues indicates that ORs are likely differentially activated by the characteristic vibrational frequencies of functional groups on odorants they engage. Single odorants activate more than one OR, albeit to different extents [(Hallem et al., 2004; Yao et al., 2005; Hallem and Carlson, 2006; Galizia, 2014; and DoOR data base (http://neuro.uni-konstanz.de/DoOR/default.html)]. In light of our data, this suggests that odorant size, shape, or hydrophobicity allows it to engage a number of distinct ORs, but activation of a particular OR, or a subset of receptors depends on the vibrational frequencies of odorant functional groups or structures, as previously suggested (Turin, 2002; Turin and Yoshii, 2003; Solov'yov et al., 2012).

Enantiomers have the same size, hydrophobicity, and functional groups, but different shape, so when insects discriminate them (Ulland et al., 2006; Sato et al., 2015), it may be based on the latter, although the majority of enantiomeric pairs smell identical (Turin and Yoshii, 2003). Because ORs must be chiral, then enantiomers may engage different receptors based on their shape differences but still activate them via the vibration-sensing mechanism previously proposed (Turin, 1996; Turin and Yoshii, 2003; Solov'yov et al., 2012).

Isotopologues do not differ in shape and functional groups, but rather in their vibrational modes. So, do they engage and activate the same ORs, or a subset of the receptors that the normal odorant engages? The similarity of EAG shapes elicited by isotopologues (Figs. 1, 2) suggests that they do not engage completely different ORs, which would likely yield EAGs of distinct shapes. Rather, isotopologues likely engage the same set of ORs, probably based on their shape, size, or hydrophobicity, but do not activate them equally, and this is reflected in the EAG amplitude differences. Because EAGs report total OR activity, traces summarize the activation and inhibition of ORs responsive to a particular odorant, with maximally activated ORs likely to contribute differentially to the EAG amplitude. Hence, each HEL isotopologue, for example, activates maximally a subset of ORs that typically engage hexanol, and the activities of these differentially activated ORs are reflected in the distinct amplitudes. Alternatively, both isotopologues activate the same exact ORs, but each to a different extent. Therefore, we propose that the isotopologue-specific EAG amplitudes reflect differences in the subsets of maximally activated ORs or the degree of activation of the same ORs.

This model makes specific experimental predictions, which are currently under investigation. Imaging of OR activity yields a specific pattern of receptors activated to different extents (Korsching, 2002; Silbering et al., 2012). The first possibility suggests that isotopologues will share the overall pattern of OR activation, but differ in the degree of activation of particular receptors. Alternatively, whereas both isotopologues will activate common receptors, each will also activate additional unique ORs. While this manuscript was in revision, independent experiments on bee antennal lobe glomeruli, imaged while the animals were exposed to deuterated isotopologues of different odorants, were reported (Paoli et al., 2016). Specific glomeruli in the same animal were preferentially activated by one of the isotopologues, while others exhibited the same response to both isotopologues, and some were inhibited by one of a pair of isotopologues. The responses of these specific glomeruli were conserved in all individual animals tested, strongly indicating that as in *Drosophila*, bee ORs discriminate isotopologues. Since isotopologues generate distinct activation maps that include differential activation, inhibition, or equal activation of specific bee ORs, it is likely that a similar situation occurs in *Drosophila*. This suggests that both types of responses presented as alternatives above are likely to be uncovered in *Drosophila* as well. In summary, the experiments detailed herein and the complementary imaging experiments in bees demonstrate that isotopologue discrimination underlies a general property of their olfactory systems, strongly indicating in turn that molecular vibrations contribute to smell in insects.

Whether molecular vibrations are a component of vertebrate and human olfaction is contested at the moment (Block et al., 2015a, b; Turin et al., 2015; Vosshall, 2015). Behavioral experiments in humans indicated that subjects were able to discriminate cyclopentadecanone isotopologues (Gane et al., 2013), but not those of the much smaller ACP (Keller and Vosshall, 2004; Gane et al., 2013). In contrast, differential activation by isotopologues was not reported when mouse ORs were expressed in cultured human kidney cells (Block et al., 2015a, b). Although perireceptor effects are not important in fly isotopologue discrimination, they may in fact be required for humans (Schilling et al., 2010), and clearly such mechanisms are lacking in tissue culture. Given that vertebrate ORs are GPCRs, while insect ORs are not, direct measurements of OR activity *in situ* akin to these described herein and in bees (Paoli et al., 2016) are essential to address the issue definitively. A mouse or other vertebrate model is likely to serve that purpose, and conversely fly dimeric ORs expressed in a heterologous system will complement these experiments.

The notion that, like hearing and color vision, smell is at least in part a spectral sense is indeed attractive, but additional experiments are needed to establish it fully, and to explore and define its parameters with precision. We have shown in *Drosophila*, and it has been reported for bees (Paoli et al., 2016), that at least for some odorants one component of OR activation is conferred by molecular vibrations. Molecular size, shape, or hydrophobicity of the odorant molecules are also likely involved, in combination with molecular vibrations or alone, for selective OR activation. Odor character has been proposed to be determined largely by molecular vibrations, while odor intensity by its shape (Turin and Yoshii, 2003). To address this working hypothesis requires electrophysiological recordings from single *Drosophila* ORs. However, given our data and those from bees (Paoli et al., 2016), the utilization of molecular vibrations to maximally activate specific receptors from those engaged by odorants based on the shape or size alone would provide additional selectivity and specificity. We do not know at the moment whether molecular vibrations are essential for the activation of all ORs in *Drosophila* and bees, but clearly they are for some. It would appear then that olfaction in *Drosophila* combines both chemical (functional groups, shape) and spectral (vibrations) components, and may be evolutionarily intermediate between the purely spectral senses (vision and hearing) and a purely chemical one (taste).

The exact mechanism that selectively activates ORs based on the molecular vibrations of odorant molecules is unclear at the moment. Because electron transfer in biological systems is not uncommon, theoretical models suggesting an electron tunneling mechanism for vibrational sensing (Turin, 1996; Brookes et al., 2007; Bittner et al., 2012; Solov'yov et al., 2012) are likely. However, such models are still incomplete because the structure of insect and vertebrate ORs is currently unknown; therefore, it is difficult to estimate the reorganization energy of the putative odorant-interacting sites that would be permissive to electron tunneling. Very recently, however, Reese et al. (2016) suggested that reorganization of molecular vibrations at the OR binding site are minimal and hence cannot suppress the proposed electron transfer mechanism (Brookes et al., 2007) of the odorant vibrational frequencies, as has been suggested before (Block et al., 2015a, b). Additional theoretical and experimental considerations on the vibration-sensing mechanism can be found in several recent publications (Vosshall, 2015; Paoli et al., 2016; Reese et al., 2016).

Although our results do not address these models directly, the identification of *Drosophila* ORs differentially responsive to one isotopologue, as in bees (Paoli et al., 2016), may offer an experimental approach. If the mechanism of vibrational detection requires electron movement, then amino acids acting as donors and acceptors may characterize ORs responding differentially to isotopologues of a particular odorant. Our evidence that isotopologue detection is broadly conserved within the genus will likely help to define such amino acids, which will be essential for future functional physiological, imaging, and behavioral experiments in single receptors *in vivo*.

## Synthesis

The decision was a result of the Reviewing Editor Darcy Kelley and the peer reviewers coming together and discussing their recommendations until a consensus was reached. A fact-based synthesis statement explaining their decision and outlining what is needed to prepare a revision is listed below. The following reviewer(s) agreed to reveal their identity: Joel Mainland</synthesis-intro>

This paper addresses the vibrational theory of olfaction using electrophysiological recordings from the antennae of several Drosophilid species [electroantennogram or "EAG" response] exposed to a range of odorant isotopes ["isotopomers"] and find that EAG amplitude, but not kinetics, differs in response to hydrogen and deuterium odorants. Inhibition of enzymes that catabolize odorants has no effect on the isotope response. The authors conclude that in vivo, isotope differentiation is not a perireceptor event, but likely occurs at the receptor itself, and that Drosophila readily distinguish odorants based on their molecular vibrations, which is the thesis of the vibrational theory of olfaction [VTO]. Given the controversy surrounding this theory, and the substantive nature of the findings, both reviewers agree that the paper could be an important contribution to the field.

The major concern is whether molecular vibration per se rather than trace impurities in the compounds chosen to represent different vibrational effects are responsible for the results obtained. This issue arises from theoretical considerations as well as observations in mammals that batches of the same compound from the same manufacturer can differ in evoked activity in mammals (humans, dogs, rats).

Here some additional data and a few specific control experiments could effectively address concerns about trace impurities:

A puzzling results from this paper is that the authors found no difference in response to 2 or 3 versus up to 13 deuterium atoms in a given molecule, but did observe a difference from all-H to partial or full deuteration. If the vibrational theory is indeed correct electron tunneling should be more efficient with a larger percentage of C-D bonds so the result is surprising. Regardless of the percentage of the C-H bonds replaced by C-D bonds, at lower concentrations the difference in the effect of isotopomers vanishes. It would be helpful if the authors could provide full details for the dilution experiments, so the reader could compare the EAG response of heavily and lightly deuterated isotopomers as dilution progresses. For example with serial dilution, does d13-1-hexanol show precisely the same decrease in amplitude of response as d2-1-hexanol, the latter having only 15% of the C-D bonds of the d13 isotopomer?

Another source of concern about impurities is that the deuterated and nondeuterated compounds come from different companies. The authors clearly show that almost all commercial samples contain small levels of contaminants (e.g., less than 1%) and the amounts, and presumably identities of such contaminants based on retention time, differ from sample to sample. Since "thousands of odorants are discriminated with exquisite specificity by olfactory receptors", then it can be argued that it is the batch to batch difference in trace contaminants between the different isotopomers that is the basis for the observed results rather than the presence or absence of C-D and C-H bonds. While the authors note that benzonitrile isotopomers give identical responses, which seems to be contrary to the notion that impurities can make samples distinguishable, it can be argued that not all impurities are equally potent. It is possible that the benzonitrile impurities do not substantially activate the different receptors sufficiently to lead to a difference at the concentrations studied.

This factor might explain that differ from those published by Gane and Turin who found that their compounds required large numbers of deuteriums (e.g. 28 C-D bonds) for detection. The batch of deuterated isotopomers of acetophenone and of 1-hexanol might have had a different set of impurities than the undeuterated analogs manufactured by different companies. To address this concern, several different samples of acetophenone and 1-hexanol from different sources, in the same range of purity as the samples previously studied (e.g. >99.6% pure), could be used to determine if the Drosophila responses are within the experimental results from the previous runs.

Also in this vein, it is entirely possible (though perhaps unlikely) that a compound below 0.1% in the GC will have a profound impact on the EAG, much as beta-Damascenone and beta-Ionone have a huge odor impact on rose odor despite being present at very low levels. It is also possible that an impurity in the d6-Benzaldehyde will have a high efficacy while the impurities in the h-Benzaldehyde will have little or no efficacy. A more convincing experiment would be to record EAG in response to the odor coming off the GC at the correct retention time as in the Gane et al., 2013 paper.

Minor points:

1) Introduction: "A vibration-sensing component was also reported recently for human olfaction." This implies that a mechanism was found for sensing vibration in humans. The Gane paper found that humans could discriminate isotopomers, but did not propose a mechanism. That paper also did not rule out all the possibilities that were controlled for here.

2) "Collectively there does not appear to be a correlation with isotope rise times and the observed amplitude differences"

Please quantify this.

3) Please report F and p-values for the ANOVAs supporting figure 6

4)The general argument is that the C-D stretch will activate a receptor that responds to the relevant vibration, while the C-H stretch will not. The Drosophila generalize to the nitrile in the Franco paper because both the C-D stretch and the nitrile will activate this receptor. Here they argue that the deuterated and undeuterated molecules will activate the same sets of ORs, but differ in which is maximally activated, which is a relatively large departure from previous ideas. The section of the discussion where the authors discuss the idea that both isotopes will activate the same sets of ORs, but differ in which is maximally activated is however somewhat confusing. The vibrational theory is generally presented as a case where the receptor detects vibrations in a given range. If the molecule doesn't vibrate in the C-D range, then the receptor would not be activated. Is this a break with the "swipe-card" model?

5) The authors should mention that these results are limited to the Drosophila and do not address the plausibility of the vibration theory in mammals.

6) Some additional information about why the vibrational theory is controversial would be helpful for the reader. This might include that given the prevalence of C-H bonds in protein environments, competing effects of nonodorant quantum vibrational modes should be considered. In addition, when a variety of mammalian ORs highly responsive to certain ligands are presented with variously deuterated isotopomers of these ligands no difference is seen, in opposition to predictions of the vibrational theory (see PNAS 2015 112(21) 6525-6526,and PNAS 2015 112(25) E3154 &amp; E3155.)

7) Olfactory receptors can discriminate enantiomers, as can many animals/insects perceptually [Journal of Chemical Education, 2010, 87(6):598-603]. The vibrational spectra of enantiomers are identical so the VTO cannot alone explain this key observation. To be valid, the VTO needs theoretical support. Some further discussion is warranted particularly for the issue of why both shape and vibration might account for specificity.

8) The language used in this manuscript to describe the different isotopically-labelled compounds as "isotopes" is sloppy and should be corrected. The correct terminology is "isotopically substituted compounds" [See: http://www.acdlabs.com/IUPAC/nomenclature/93/r93_651.htm http://www.acdlabs.com/iupac/nomenclature/93/r93_645.htm http://www.acdlabs.com/iupac/nomenclature/93/r93_650.htm ] according to the International Union of Pure and Applied Chemistry (IUPAC). The nuclides hydrogen and deuterium are isotopes; isotopically labeled compounds are compounds in which one or more normal nuclide, such as H, is replaced by a different one, such as D.
